# Transgenerational effects in asexually reproduced offspring of *Populus*

**DOI:** 10.1371/journal.pone.0208591

**Published:** 2018-12-06

**Authors:** Sumitra Dewan, Pieter De Frenne, An Vanden Broeck, Marijke Steenackers, Kristine Vander Mijnsbrugge, Kris Verheyen

**Affiliations:** 1 Forest & Nature Lab, Department of Environment, Ghent University, Gontrode, Belgium; 2 Research Institute for Nature and Forest (INBO), Geraardsbergen, Belgium; Technical University in Zvolen, SLOVAKIA

## Abstract

The response of trees to a changing climate can be affected by transgenerational phenotypic plasticity, i.e. phenotypic variation that is conserved and transferred to the offspring. Transgenerational plasticity that is influenced by epigenetics (heritable changes in gene function that do not result from changes in DNA sequence) during both sexual and asexual reproduction are of major relevance for adaptation of plants to climate change. To understand the transgenerational effects on the responses of vegetatively propagated poplar (*Populus deltoides* and *P*. *trichocarpa*) ramets (cuttings) to a changing environment, we tested whether the temperature and photoperiod experienced by the mother trees (genets) persistently affects the phenology of the cuttings grown in a common environment. We weekly monitored the bud phenology of the cuttings collected from the parent trees that have been growing across Europe along a >2100 km latitudinal gradient for at least 18 years. In addition, we asked whether there was variation in DNA methylation as measured by Methylation Sensitive Amplified Fragment Length Polymorphism (MSAPs) in the clones due to the different environmental conditions experienced by the parent trees. Our results indicate a transgenerational effect on bud phenology in the asexually reproduced offspring (vegetative cuttings). The temperatures experienced by the parent tree clones (from different geographic regions) altered the bud flush of the cuttings in the common garden. However, no significant epigenetic variation was detected in the cuttings of the parent trees within single genotypes growing under different climates. In sum, our results show that trees have the potential to respond to rapid climate change but the mechanism behind these changes needs to be further investigated by more powerful molecular methods like whole-genome bisulphite sequencing techniques.

## Introduction

Phenotypic plasticity is an important mechanism for trees to cope with climate change and heterogeneous environments [1]. Bud phenology (here defined as the timing of bud flush and bud set) in particular is a very important phenotypic trait to assess plant responses to climate change since it controls the growing period available to plants. Along with other phenotypic traits, it received increased attention in the light of global warming [2–5]. Bud phenology can be influenced by global warming and interactions between the climate and photoperiod [4, 6, 7]. However, the phenology of tree species does not show simple linear responses to warming temperatures [8]. In the case of a latitudinal transfer from south to north, i.e., if plants experience colder than optimal temperatures, longer time would be required for the duration of bud formation and the cessation of growth can be delayed [6]. One component of phenotypic plasticity is transgenerational effects when the parent’s environment influences offspring responses to environmental conditions independent of genetic changes [9, 10], which is also known as transgenerational phenotypic plasticity (TGP). It is particularly important for tree responses to climate change [10]. Most former studies (on plants) have focused on transgenerational (particularly maternal) effects of sexually reproduced offspring, examining the effects of the maternal environment on seed germination and flowering time [11–13]. Various studies, by comparing the performance of the progeny in maternal and non-maternal environments, have shown that maternal effects are likely adaptive [12, 14]. However, in heterogeneous environments where the progeny environment is hard to predict, the phenotypes with low variance of fitness get a selective advantage which is also known as bet hedging phenomena and in such conditions the advantage of parental effects might not be observed [15]. Although thus far this effect has been studied mainly in non-clonal plants, transgenerational plasticity is also applicable to asexually generated progeny [14].

Asexual reproduction lacks the variation-generating mechanisms of meiotic recombination and segregation, which reduces the potential for genetically based adaptation [16]. Nevertheless, many clonal species persist and successfully expand in a range of different environments. Some of the most successful invasive plant species are clonal [17, 18]. Clonal individuals have the advantage of resource sharing (such as water, carbohydrates and mineral nutrients) between the connected ramets, an effect known as clonal integration which is probably one reason of their invasion success [18]. Furthermore, epigenetic variation might contribute to adaptation of asexual plants in a wide range of, particularly stressful, environments [19]. It is suggested that, given the absence of meiotic resetting of epigenetic modification in clonal reproduction, vegetative offspring can inherit epigenetic information of previous environmental interactions from the maternal ramet [16]. Transgenerational effects were observed in clonal offspring of *Festuca rubra* and *Trifolium repens* and some transgenerational effects in clonal plants of *Trifolium repens* were reported as adaptive [20, 21]. DNA methylation, the addition of a methyl group to one of the four bases in the DNA molecule (usually cytosine), is recognized as one of the prime epigenetic mechanisms to correlate with gene expression and might play an important role in transgenerational effects. However, the processes behind transgenerational effects are not well understood [14, 19, 22]. Yet, transgenerational effects in clonal plants are important for forest management, reforestation programmes and nurseries, because many tree species (e.g. poplars, willows and many fruit trees) are reproduced vegetatively by means of cuttings or grafting.

Poplars (*Populus* sp.), member of the *Salicaceae*, have a wide distribution in the world and can easily be propagated vegetatively. The species therefore serves as an ideal model to study responses of asexually reproducing plants to climate change. *Populus* is a fast growing genus important for biomass production, and can produce 70–105 tons of aboveground biomass per hectare after 10–15 years [23]. The hybrid poplars are also planted widely in Europe for wood production, windbreaks and soil protection [24, 25].

Here we set up a common garden experiment to assess bud phenology of cuttings collected from genets growing in different climatic environments (further referred to as: parent trees) and representing five different clones of *Populus trichocarpa* × *P*. *deltoides* and *Populus trichocarpa* (further referred to as: genotypes). Parent trees representing a single genotype were grown across a latitudinal gradient of >2100 kilometres (corresponding to a 4.9°C temperature difference and different photoperiods of up to 3.5 hours), and cuttings of these parent trees were then grown in the common garden. We hypothesize a transgenerational effect of temperature experienced by the parent trees on bud phenology of the vegetatively (by stem cuttings) produced offspring within genotypes. Furthermore, we investigated DNA methylation variation as a potential epigenetic mechanism for transgenerational effects (in this case, epigenetic variation in the generations of cuttings) using Methylation Sensitive Amplified Fragment Length Polymorphism (MSAP) analysis [26]. The aim of the molecular part of this study was to look for significant natural variation in genome-wide DNA methylation patterns within individuals plants of a single clone (genotype) and vegetatively propagated from parent trees with different histories (that is, grown in contrasting macroclimates across Europe), that lasted after growing for four years in a common environment.

## Materials and methods

### Description of the clones

Between the 1980s to 1990s cuttings of five hybrid poplar genotypes namely Beaupré, Raspalje and Unal (all *Populus trichocarpa* × *P*. *deltoides*), Fritzy Pauley (*P*. *trichocarpa)*, and Trichobel (*P*. *trichocarpa* × *P*. *trichocarpa*) were produced from adult trees under the renowned poplar breeding programme at the Institute of Forest and Nature (INBO), Geraardsbergen in Belgium. The adult trees that resulted from the original seedlings selected in the breeding programme (further referred to as ‘provenance trees’) remained in Grimminge, Belgium and were not transferred. Asexually reproduced offspring of the provenance trees (onwards referred to as ‘parent trees’ or ‘genets’) were taken as cuttings and planted in Spain (near Madrid), Italy (Casale Monferrato), France (Saint-Usage, Beuxes, Gueméne Penfao) and Sweden (Uppsala) to establish stool beds for the purpose of tree breeding (Table 1). Later, new stool beds were established in the vicinity of the old stool beds from cuttings of the former beds.

**Table 1:** Background information of the study sites across Europe where poplar trees were transplanted and sampled.

Country	Site	Latitude (°)	Longitude (°)	Elevation (m)	MJanT^a^ (°C)	MJulyT^b^ (°C)	MAT^c^ (°C)	DL^d^ (h) 1 Jan.	DL^d^ (h) 1 May	Cuttings planted (year)
France	Beuxes	47.09	0.18	43	5.42	19.17	11.99	8.36	14.25	1994
Spain	Madrid	40.68	-4.10	988	3.46	21.98	11.92	9.14	13.79	1996
France	Gueméne Penfao	47.63	1.89	139	3.54	19.04	11.04	8.29	14.30	1985
Belgium	Grimminge	50.78	3.93	28	3.96	18.18	10.86	7.81	14.58	Provenance trees
Italy	Casale Monferrato	45.08	8.30	161	1.49	18.94	9.46	8.63	14.10	1982
France	Saint-Usage	47.08	5.24	178	0.69	18.22	9.10	8.36	14.25	1994
Sweden	Uppsala	59.86	17.64	18	-0.87	16.97	7.01	5.71	15.70	1990
Belgium	Geraardsbergen	50.78	3.88	29	3.96	18.18	10.86	7.81	14.58	Common garden

^a^MJanT- mean monthly temperature for January,

^b^MJulyT- mean monthly temperature for July,

^c^MAT- mean annual temperature

^d^DL-Day length

### Sample (cuttings) collection

Between February and April 2014 before the start of bud burst, we collected 817 one year old cuttings of 22 cm length of the above mentioned five *Populus* genotypes growing at seven sites; including the local site of the provenance trees in Grimminge, Belgium (Table 1). Not all five genotypes were present in all the seven sites and the number of cuttings of each genotype was not evenly distributed either due to mortality or bud damage. Each cutting was measured (collar diameter) and planted in individual 5 L pots containing standard potting soil (Saniflor pro, NPK 12-14-24) and monitored in a common garden outside the Institute of Forest and Nature (INBO), Geraardsbergen, Belgium (Table 1). The cuttings were regularly irrigated and fungicide (‘Caddy’- Cyproconazole) was applied two times on 5/05/2015 and 26/08/2015 during the growing season in the common garden. It was known that the application of the above fungicide (Cyproconazole) influenced the gene expression of the fungal pathogens, but the effect on the DNA methylation or on the gene expression of the plant community is yet to be discovered [27].

### Observation of growth and bud phenology

We quantified autumn phenology (bud set) in 2014 and both spring and autumn phenology (bud burst and bud set) in 2015. The bud burst and bud set was assessed once a week (starting on 17 March 2015 i.e. Day of the year (DOY) 76 for bud burst and on 13 August 2014 (DOY 225) and 21 August 2015 (DOY 233) for bud set) until all the cuttings completed bud burst and bud set. The bud set and bud burst was monitored by scoring each plant according to the method of [28, 29] with a few modifications as detailed in S1 Table. We measured the height (cm) of the cuttings in December 2014 after end of the growing season.

### Temperature and day length data

The mean 1982–2014 annual temperature (°C) (MAT), mean monthly temperature for July (MJulyT), mean monthly temperature for January (MJanT) at the seven sites where the clones had been growing were calculated using the *RFc* package in R version 3.3.3 [30]. We chose mean July and January temperatures to represent the warmest and coldest month of the year, because it has been shown before that minimum and maximum temperatures, rather than mean annual temperatures, better predict leafing, flowering and growth of several plant species [31–33]. Day length on 1 May (DL1May) and day length on 1 January (DL 1Jan) at all seven sites were calculated according to [34].

The growing period of each plant for 2015 was calculated by counting the number of calendar days between complete bud burst (bud burst score equal to 5) and complete bud set (bud set score equal to 0).

### Determination of DNA methylation

#### Plant materials and sample collection

DNA methylation variation was studied within the poplar clones growing in the common garden experiment. On 17 April 2017 (that is, after four growing seasons in the common garden), 54 leaf samples (young, freshly developed leaves) were collected from one year old stem of the cuttings in the common garden (S2 Table). Just after collection, fresh leaves were stored in silica gel until DNA extraction.

#### DNA samples

DNA samples were obtained from the same plant tissue and collected at identical developmental stage (newly expanded, fully-grown leaves). Total genomic DNA was extracted from these leaves with the QuickPickTM Plant DNA kit (Isogen Life Science, De Meern, Nederland). The integrity of the DNA was assessed on 1.5% agarose gels, and DNA quantification was performed with Quant-iT PicoGreen dsDNA Assay Kit (Life Technologies) using a Synergy HT plate reader (BioTek).

#### Verifying clone (genotype) identity with microsatellite markers

Twelve nuclear microsatellite loci (SSRs) were used to verify the identity of the genotypes. We selected SSRs that were found useful for the identification of *Populus* genotypes in former studies [35, 36]. PCR products were run on an ABI 3500 analyzer with the GeneScan-600 LIZ size standard and analyzed using GeneMapper 4.1 (Thermo Fisher Scientific). Details on microsatellites and PCR-conditions are given in S3 Table.

#### Methylation-sensitive Amplified Length Polymorphism

The Methylation Sensitive Amplified Length Polymorphism (MSAP) method, a modified version of the Amplified Fragment Length Polymorphism (AFLP) DNA fingerprinting technique [37], was adapted from [26]. Briefly, we initially tested 32 primer combinations on a subset of 16 samples using two sets of restriction and ligation reactions. Seven combinations of *Eco*RI (labelled primers) / *Hpa*II—*Msp*I primers (S4 Table) were selected for the MSAP analysis of the total 54 samples, based on clarity and reproducibility of amplified bands and the presence of polymorphism. Eleven samples were replicated, starting from the same leaf sample and two different DNA-extractions to assess reproducibility. PCR amplicons were fluorescently labeled with one of two dyes: NED and VIC, and were run in simplex on an ABI 3500 analyzer with the GeneScan-600 LIZ size standard (Thermo Fisher Scientific). The *Eco*RI—*Msp*I and *Eco*RI—*Hpa*II DNA fingerprinting profiles were processed per primer combination. Only fragments ≥150 bp in size were considered to reduce the potential impact of size homoplasie [38]. The genotyping error rate was estimated for each primer combination according to [39] and based on the 11 replicates.

### Statistical analysis

#### Bud phenology

All statistical analyses were performed in R version 3.3.3 [40]. Linear mixed effects models (*lmer* function in the *lme4* package in R) were used to analyse the relationship between phenology (bud burst and bud set) and temperature (MJanT, MJulyT, MAT), stem diameter (mm) and day length (DL1May and DL1Jan) of the seven sites (parental environment) where the parent trees of the different genotypes were growing [41]. The number of cuttings (ramets) per genotype collected at each of the seven parental environments, however, was not evenly distributed (S2 Table). So, instead of one combined model with *genotype* as random factor, we applied the above model for each genotype separately to reduce the bias of small vs. large sample sizes in the model. Moreover, the phenological response can also differ depending on the genotype. Since the relationship between the phenology and temperature variables may change over the gradual progress of bud set or bud burst [42], we applied the same linear mixed effects model for each observation day (days of the year, DOY) separately for 2014 and 2015. We used *site* as a random effect in the models. The weighted mean of the slopes of all genotypes for each temperature variable was then calculated by using the slopes from the mixed models by bootstrapping 500 times (*boot* function in the *boot* package in R) [43]. Similarly, we used linear mixed effect models to analyse the relationship between the length of the growing season and temperature variables (MJanT, MJulyT, MAT) and day length (DL1May and DL1Jan) using *site* as random effect for each genotype. Then, we calculated the weighted mean of the slopes from the models again by using bootstrapping as above. We also analysed the relationship between height (cm) of the cuttings after one growing season that was in 2014 and total days (day) needed to open the bud (time to bud burst score 5) starting from 17 March (= observation day 1) in 2015 using Generalised Linear models with Poisson error distributions.

#### DNA methylation

We used GeneMapper v3.7 (Thermo Fisher Scientific) for the sizing of the DNA fragments (raw data) and the RawGeno v 2.0 R CRAN package [44] for automatic scoring of the variation in the sized fragment patterns and to transform the fragment profiles into a binary character matrix, using 0 or 1 to define the absence or the presence of a specific DNA band, respectively. The *msap* was used to assess the cytosine CpG methylation profile of CCGG motifs for each sample and to analyze the data [45]. The presence of both *Eco*I / *Msp*I and *Eco*RI / *Hpa*II products (pattern 1/1) denotes an unmethylated state, the presence of only one of the EcoRI / HpaII (1/0) or EcoRI / MspI (0/1) products represent methylated states (hemimethylated or internal C methylation) and absence from both *Eco*RI / *Msp*I and *Eco*RI / *Hpa*II products (0/0) is considered as an uninformative state, as it could be caused by either fragment absence or hypermethylation [45]. The epigenetic state scoring error rate was estimated for each primer combination from discordant scores in *Msp*I and *Hpa*II profiles of 11 individuals that were processed twice from different DNA extracts. Following the procedure in [46], every loci was then classified as either Methylation-susceptible loci (MSL) or Nonmethylated loci (NML), depending on whether the observed proportion of methylated states across all samples exceeded the estimated error rate. Only samples without missing data for the seven primer combinations and for both enzyme combinations are included in the analysis. This resulted in 52 samples analyzed (replicated samples excluded) for in total 233 MSAP-fragments.

The analysis was performed by grouping the clones per country. The amount of genetic and epigenetic variation was estimated using the Shannon diversity index (S). The epigenetic differentiation between groups (φ_ST_) was tested using analyses of molecular variance (AMOVA) based on 1000 permutations. A Mantel test was performed to obtain the correlation between MSL and NML and to shed light on how much epigenetic variation was influenced by the genetic background. The genetic (NML) and epigenetic (MSL) structure was assessed by a principal coordinates analysis (PCoA).

## Results

### Bud burst, bud set and growing season

**Fig 1:**
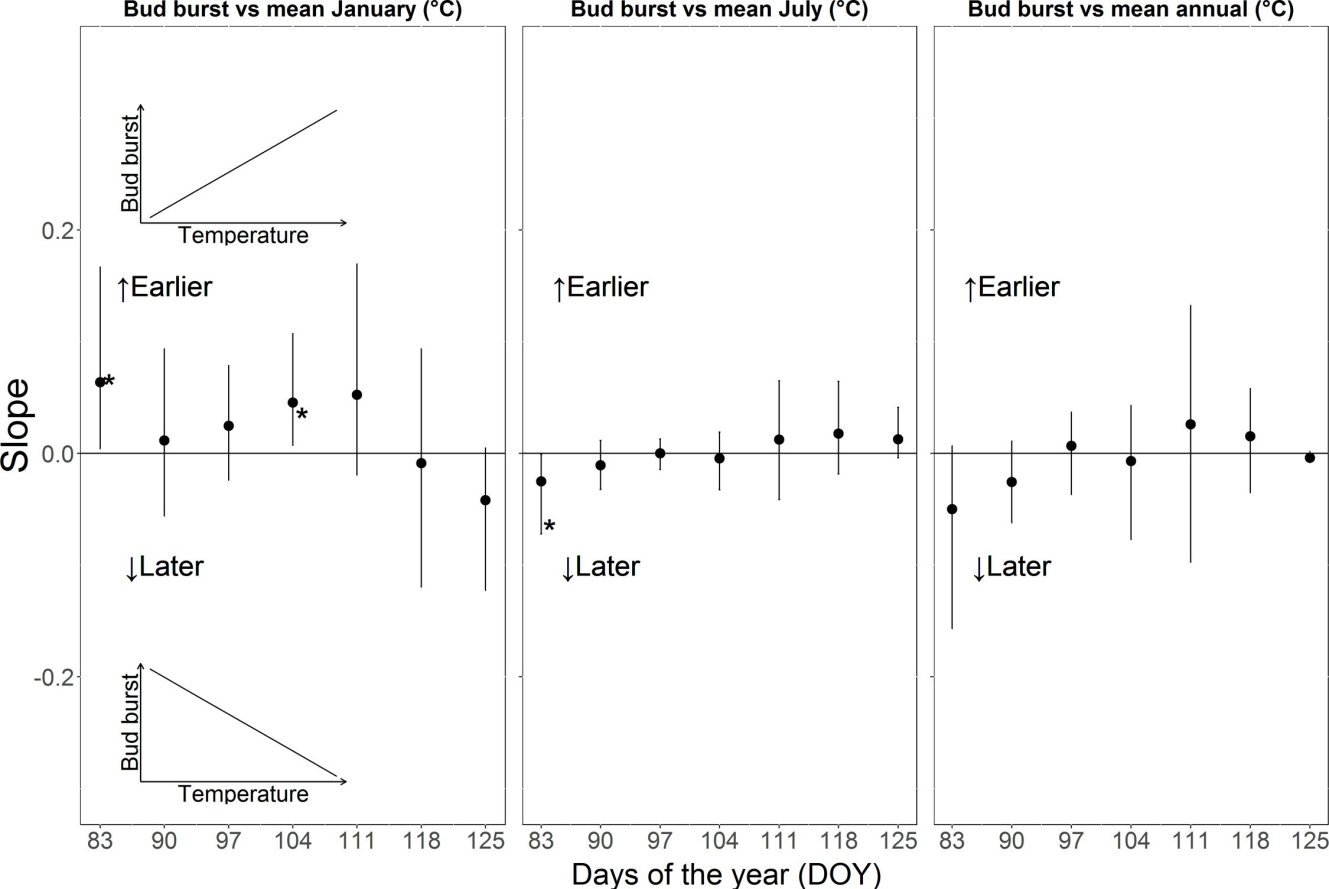
Mean weighted (bootstrapped) slopes of the relationship between the mean bud burst score in 2015 and mean January, July and mean annual temperatures experienced by the parent trees. Error bars denote 95% confidence intervals (upper and lower) across the 500 bootstrapped values. Significances at the 95% level are denoted by *. **‘Earlier’** means that buds burst earlier with increasing temperatures and **‘Later’** means that buds burst later with increasing temperatures.

**Fig 2:**
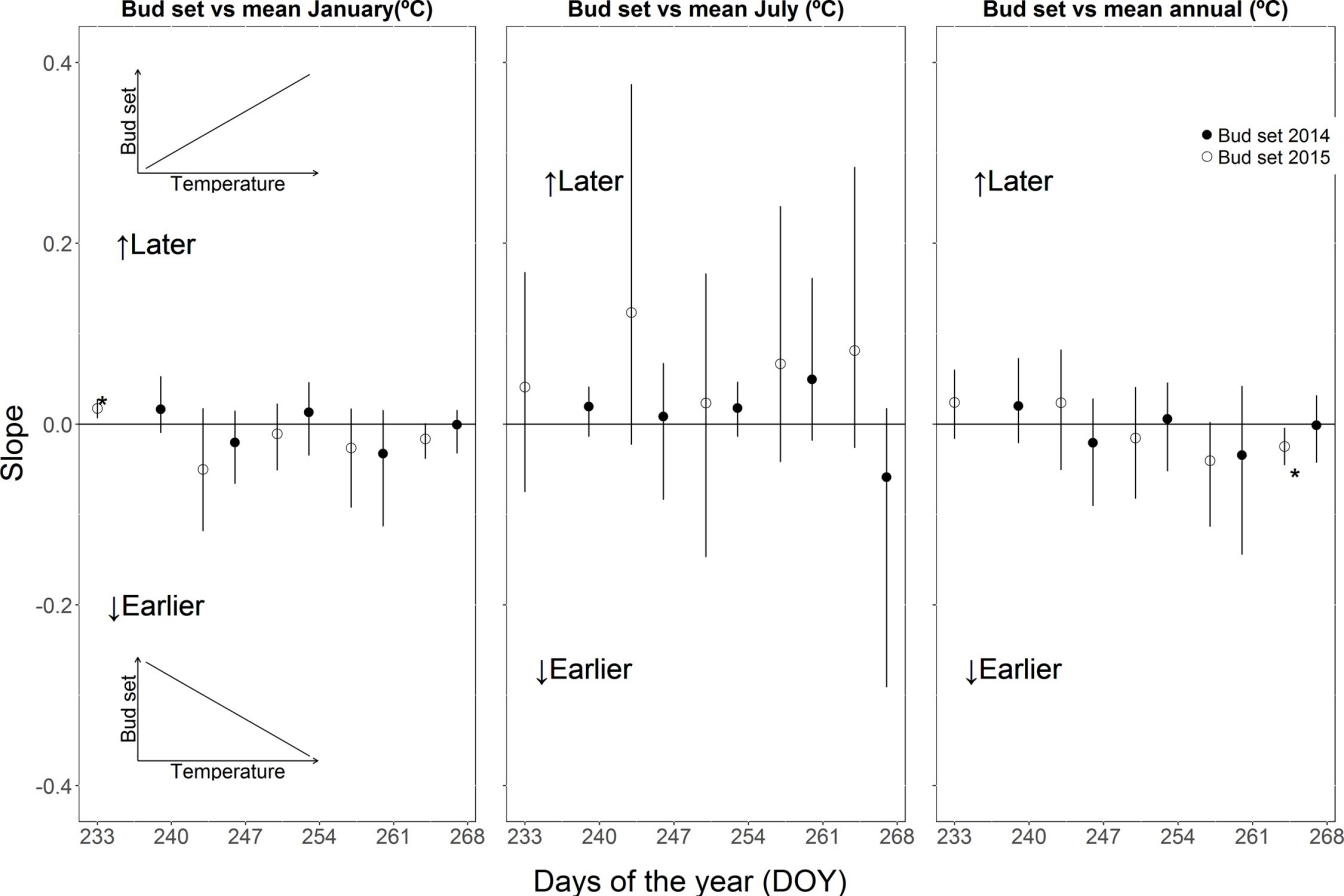
Mean weighted (bootstrapped) slopes of the relationship between the mean bud set score in 2014 and 2015 and mean January, mean July and mean annual temperatures experienced by the parent trees. Error bars denote 95% confidence interval (upper and lower) across the 500 bootstrapped values. Significances at the 95% level are denoted by *. ‘Earlier’ means that buds set earlier with increasing temperatures and ‘Later’ means that buds set later with increasing temperatures.

We observed earlier bud burst (that means a higher bud burst score) in cuttings in 2015 where the parent tree experienced warmer mean January, July and mean annual temperature across almost all poplar genotypes, which was presented by positive slope of the relationship between temperature and mean bud burst score (Fig 1). The plots showing the relationship between mean annual temperatures (MAT) experienced by parent trees and mean bud burst score of the cuttings of four genotypes on day of the year 83 are available in S1 Fig. Taller plants exhibited significantly earlier bud burst in the second growing season (S2 Fig). The cuttings also set buds earlier (that means lower bud set score) with warmer January and mean annual temperatures in parental environment, which was indicated by a negative slope of the relationship between temperature and mean bud set score (Fig 2). The significant difference in bud set was observed only on one day during the second growing season. We did not observe any consistent relationship between bud burst and day length (1 May and 1 January) of the sites where the parent trees have been growing (S3 Fig). However, the day lengths on 1 May and 1 January of the growing sites of the parent trees had a significant influence on bud set in 2014 (only on day of the year 247), but in the following year no significant effect was observed (S4 Fig).

The results of linear mixed effect models for each genotype on each observation day (DOY) for budburst in 2015 and bud set in 2014 and 2015 can be found in S5 and S6 Tables.

**Fig 3:**
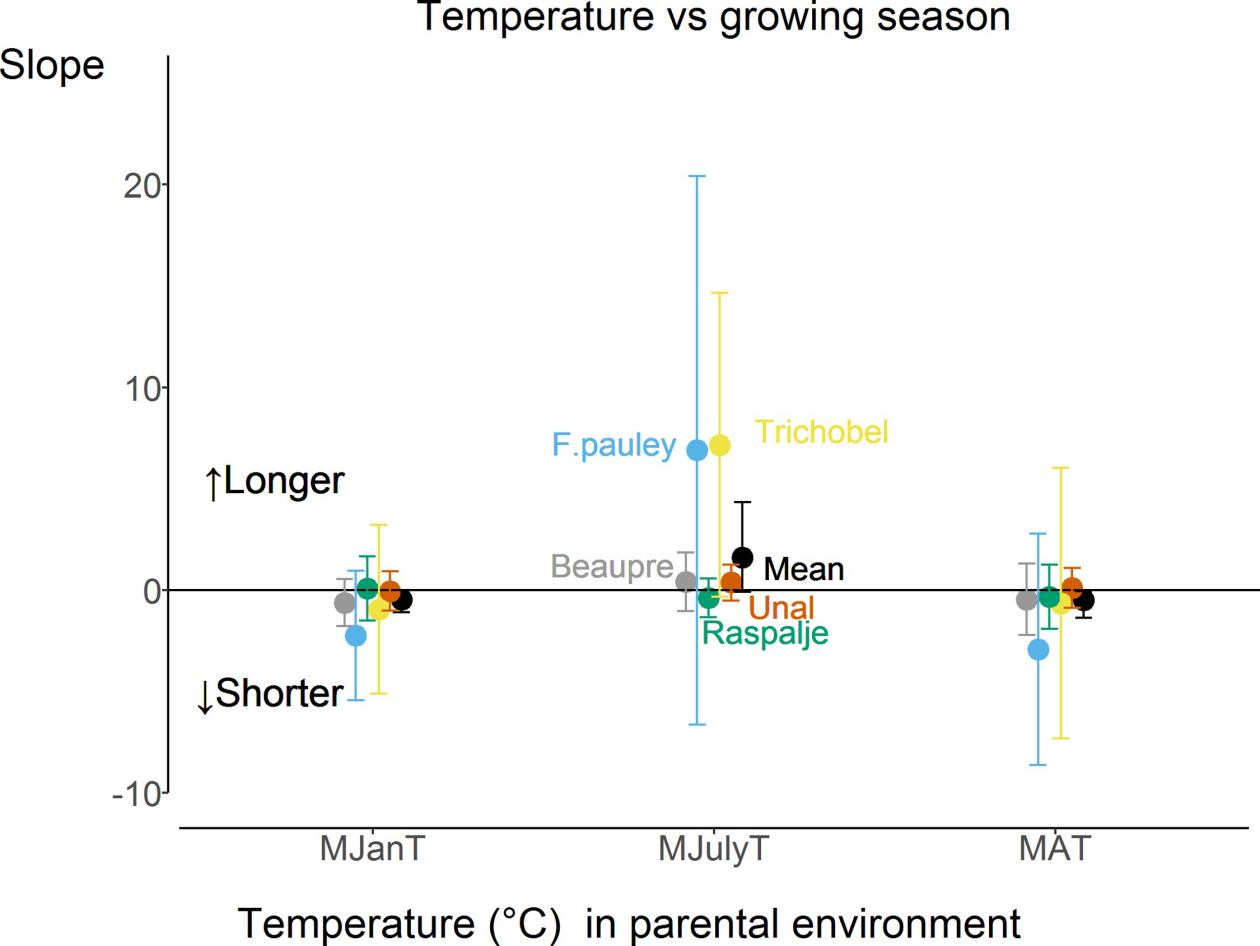
Slopes of the relationship between the length of growing season of five genotypes (Beaupre, Fritzy Pauley, Raspalje, Trichobel and Unal) in 2015 and mean January, July and annual temperatures experienced by the parent trees. Mean weighted (bootstrapped) slopes are indicated by black points and error bars denote 95% confidence intervals (upper and lower) across 500 bootstrapped values. Error bars denote 95% confidence intervals. ‘Shorter’ means that growing season is shorter with increasing temperature and ‘Longer’ means that growing season is longer with increasing temperature.

Parental temperature also significantly affected the length of the growing season of poplar cuttings probably due to earlier bud set. The length of the growing season was significantly shorter with warmer mean annual temperature of the translocated sites across all the genotypes (the weighted mean = -0.509, upper and lower confidence intervals were -0.07046 and -1.37171 respectively across 500 bootstrapped values) (Fig 3). Remarkably, there was no effect of day length on the length of the growing season (S5 Fig).

### Clone verification and DNA methylation

The clone identification was confirmed by the results of the microsatellite analysis (S3 Table). The mean estimated genotyping error rate was 0.022 (S4 Table). Of these 233 MSAP-fragments, 142 were Methylation Sensitive Loci (MSL) (of which 114 (80%) were polymorphic) and 91 were Non Methylation Sensitive Loci (NMS) (70 (77%) were polymorphic). There was no higher degree of epigenetic variation compared to genetic variation (S6 Fig). In contrast, the mean Shannon’s diversity index for MSL (Mean ± SE = 0.403 ± 0.165) was significantly lower than the corresponding figure for NML (0.494 ±0.162) (Wilcoxon rank sum test with continuity correction: W = 2631.5, P < 0.001).

The AMOVA-based estimate of epigenetic differentiation between groups was low and not statistically significant (φ_ST_ = 0.0189, p = 0.198). The Mantel test indicated a high correlation between MSL and NML (r = 0.845, p < 0.001, nperm = 1000). Similarly, the PCoA based on the methylated loci was very similar to the PCoA for the non-methylated loci, suggesting a high dependence between genetic and epigenetic variation.

## Discussion

The results of this study indicate the presence of a transgenerational effect mediated by parental environment on the bud phenology of asexually produced offspring (vegetative cuttings) of different *Populus* genotypes. Previous studies in *Populus* sp. have shown that trees from more southern locations display earlier bud burst and shoot growth cessation later in the summer compared to trees from more northern origins [28, 47, 48]. In this study, the temperatures experienced by the parent trees in different regions across Europe likely altered the bud flush of the cuttings in the common garden. Although growing in a common environment, we found a correlation of earlier bud burst with warmer January, July and mean annual temperatures of the parent environment. This indicates that the parental environment may have played an important role in altering the timing of bud burst. Processes responsible for such transgenerational effects are not yet perfectly understood, but the most important processes are likely the nutrient conditions of the parent plant and epigenetic inheritance [49]. In Norway spruce, [42] found that an epigenetic memory mechanism affects the timing of bud burst phenology and the expression of bud burst related genes in genetically identical Norway spruce epitypes (an epigenetic alteration in a gene), allowing them to adapt rapidly to a changing environment. The temperature sum experienced by the developing embryo and photoperiod conditions during embryogenesis epigenetically shift the growth cycle of the embryos, giving rise to different epitypes from the same genotype [50]. Although the latter studies provide evidence for the stable inheritance of epigenetic marks under sexual reproduction, several studies also demonstrate the stable transmission of DNA methylation from parent to clonal offspring in asexually reproducing plant species [19, 51]. Significant variability in DNA-methylation patterns as well as significant variation in bud phenology was found in Lombardy poplar, a clone of *Populus nigra* that is worldwide distributed since the 18th century [52]. However, in this study using MSAP we did not find evidence for variation in genome-wide DNA methylation patterns within plants of the same genotype and propagated from parent trees with different environmental histories. It is possible that epigenetic variation was (at least partly) erased (e.g. by epigenetic resetting resulting in reduced polymorphisms in DNA methylation) at the time the leaves were collected for molecular analysis (after almost four years in the common environment and two years after the assessment of bud phenology). In addition, the environmental clone history of the parent trees might have been too short to shape strong differences in genome-wide DNA methylation patterns in response to the environmental history. More powerful molecular techniques, such as bisulphite high-throughput sequencing techniques[53], are needed to further investigate the mechanisms behind the transgenerational effects observed in this study.

The programming to start spring growth is actually set during fall when the plant enters dormancy. In poplar, it is known that photosynthate amounts present in the stem and root in the late autumn can contribute substantially to growth and overwintering carbohydrate storage [54]. Increased growth likely influenced the bud burst time, which was suggested with our observation that taller cuttings (after one year of growth) burst buds earlier in the second growing season. Therefore, there was a possibility that the observed phenological changes were a result of developmental plasticity, which could also be considered as within-generational plasticity.

We did not observe any effect of day length on bud burst. Absent to low photoperiod-sensitivity to bud burst of two poplar species was also reported in [55]. However, the photoperiod signal was known to have an influence in reducing the temperature sum requirement to bud burst in European beech [56]. If we infer that the genotypes that were exposed to a warmer parental environment might require more accumulated heat for bud burst, then this means that bud burst of these genotypes will be delayed until reaching the sufficient heat sum [57]. Winter warming can influence the bud burst time via affecting the dormancy and the chilling requirement [58]. Advancing bud burst with winter warming, therefore suggests that chilling requirement was fulfilled earlier and temperature sum may have played important role in controlling the bud burst time [59].

We unexpectedly observed earlier bud set with warmer winter (January) and mean annual temperatures experienced by the parent trees, but the difference was observed only on one day during the second growing season. In general, the growth cessation in many temperate species was delayed with global warming and an extension of the growing season was observed [59, 60]. Following the report of [20], the change in bud phenology detected here might be influenced by origin (effect of tree provenance), parental environment (different geographic regions) and the offspring‘s environment.

Earlier bud set in our study was linked to earlier bud burst by allowing the plants an earlier start of dormancy for the chilling accumulation, which can be a trade-off between avoiding late frost damage and extending growing season. Earlier bud break translated into earlier bud set, which was also reported in *Quercus robur* L. and *Fagus sylvatica* [61]. Although, in poplar, a delayed bud set with warming was found by [6]. Equatorward transfer of a genotype, in general, shortens the period of active growth due to the reduction of the growing-season photoperiod, thereby advancing autumn growth cessation [6, 57]. Temperature can also interact with the photoperiod to alter the photoperiodic signal to growth cessation in poplar [6, 28]. Though, from the study of [59], we know that the growth cessation in many temperate species is not constrained by photoperiod. In our study, more likely the interactive effect of photoperiod and temperature had an additional influence on the earlier growth cessation [6]. The phenological changes in our study are probably not adaptive and might change over time. Adaptive plasticity is also dependent on the accuracy of the environmental cues, the degree of environmental heterogeneity and stable epigenetic marks at least within an individual’s lifetime [62].

The shortening of the growing season in cuttings of which the parent trees experienced warmer mean annual temperatures was mostly due to the earlier bud set. In some species including poplar, photosynthesis and biomass growth can be sustained until leaf senescence [54], which plays an important role in winter dormancy and spring growth by providing sufficient resources and root growth. It is likely that the earlier bud set resulted in lower growth thereby providing limited resources for winter storage and subsequent spring growth. Higher January temperatures would then fail to promote growth—even though the trees burst buds earlier—with limited biomass storage. Unlike our findings, earlier studies reported an extended growing season despite earlier spring growth and leaf senescence [61]. However, climate warming may have a positive effect on the length of the growing season as observed by many observational and climate manipulation experiments [4, 59–61, 63–65].

## Conclusion

In sum, we show that a latitudinal transfer of poplar clones resulted in contrasting phenological responses to temperature. We further our understanding of the response of trees to climate change because we do show that the parental environment can influence the phenology of the cuttings. Together with other factors such as genetic variability, variable temperature sensitivity among species, the environmental condition of parent trees needs to be taken into account to better predict the response of trees to climate change. Nevertheless, the mechanism behind the shift of the timing of bud phenology remains complex and unclear, which provides the opportunity to further investigate the mechanism behind the phenological shift due to heterogenous parental environments and whether such phenological variation is adaptive.

## Supporting information

S1 Fig The relationship between mean annual temperature (MAT) and bud burst score in the cuttings of four different clones on 2nd observation (83 days of the year-DOY) where a, b, c and d represents respectively for genotypes Unal, Raspalje, Fritzy Pauley and Trichobel.
(DOCX)

S2 Fig The relationship between number of days to bud burst in 2015 and the height of the seedlings during end of growing season in December 2014. (DOCX)

S3 Fig Mean weighted (bootstrapped) slopes of the relationship between the mean bud burst score in 2015 and day lengths on 1 May (a) and 1 January (b) experienced by the parent trees. Significances at the 95% level are denoted by *. “Earlier” means that buds set earlier with increasing temperature and “Later” means that buds set later with increasing temperature. Error bars denote 95% confidence interval (upper and lower) across the 500 bootstrapped values.
(DOCX)

S4 Fig Mean weighted (bootstrapped) slopes of the relationship between the mean bud set score in 2014 and 2015 and day lengths on 1 May (a) and 1 January (b) experienced by the parent trees. Error bars denote 95% confidence interval (upper and lower) across the 500 bootstrapped values. Significances at the 95% level are denoted by *. “Earlier” means that buds set earlier with increasing temperature and “Later” means that buds set later with increasing temperature.
(DOCX)

S5 Fig Mean weighted (bootstrapped) slopes of the relationship between the length of growing season in 2015 and day lengths on 1 May (DL1May) and 1 January (DL1Jan) experienced by the parent trees. Error bars denote 95% confidence interval (upper and lower) across the 500 bootstrapped values. ‘Shorter’ means that growing season is shorter with increasing day length and ‘Longer’ means that growing season is longer with increasing day length.
(DOCX)

S6 Fig Representation of Principal Coordinate Analysis (PCoA) for methylated (A) and non-methylated (B) loci. Samples are grouped per clone (countries are here BE = Belgium, FR = France, ES = Spain, IT = Italy and SE = Sweden). The first two coordinates (PCO1 and PCO2) are shown with the percentage of variance explained by them. Different point types represent individuals from different groups.
(DOCX)

S1 Table Description of the scoring systems of bud burst and bud set in poplar cuttings based on visual observation. (DOCX)

S2 Table Number of individuals collected and monitored from respective genotypes and country for determination of DNA mathylation and bud phenology (bud burst and set). (DOCX)

S3 Table List of the microsatellite markers used for the genotype identification. TA; annealing temperature, MP: multiplex.
(DOCX)

S4 Table Primer combinations used, number of loci and estimated genotyping error rates. (DOCX)

S5 Table The results from the linear mixed effect models (in response to temperature variables and stem diameter). NA means no data available due to 0 (zero) variance in response variable. We used *lmerTest* package to extract the *ρ* values from the linear mixed *effects* models [66].
(PDF)

S6 Table The results from the linear mixed effect models (in response to day lengths). NA means no data available due to 0 (zero) variance in response variable.
(PDF)

S1 File Bud burst data. (XLSX)

S2 File Bud set 2014 data. (XLSX)

S3 File Bud set 2015 data. (XLSX)

S4 File Data-MSL-PCoA.coor. (CSV)

S5 File Data-NML-PCoA.coor. (CSV)
