# Supplementary material for: Transgenerational effects in asexually reproduced offspring of Populus
Source: PLoS One. 2018 Dec 6;13(12):e0208591. doi: 10.1371/journal.pone.0208591 (PMC6283561; doi:10.1371/journal.pone.0208591)
Supplement: S1 Fig — The relationship between mean annual temperature (MAT) and bud burst score in the cuttings of four different clones on 2nd observation (83 days of the year-DOY) where a, b, c and d represents respectively for genotypes Unal, Raspalje, Fritzy Pauley and Trichobel. (DOCX) [file pone.0208591.s001.docx]

**
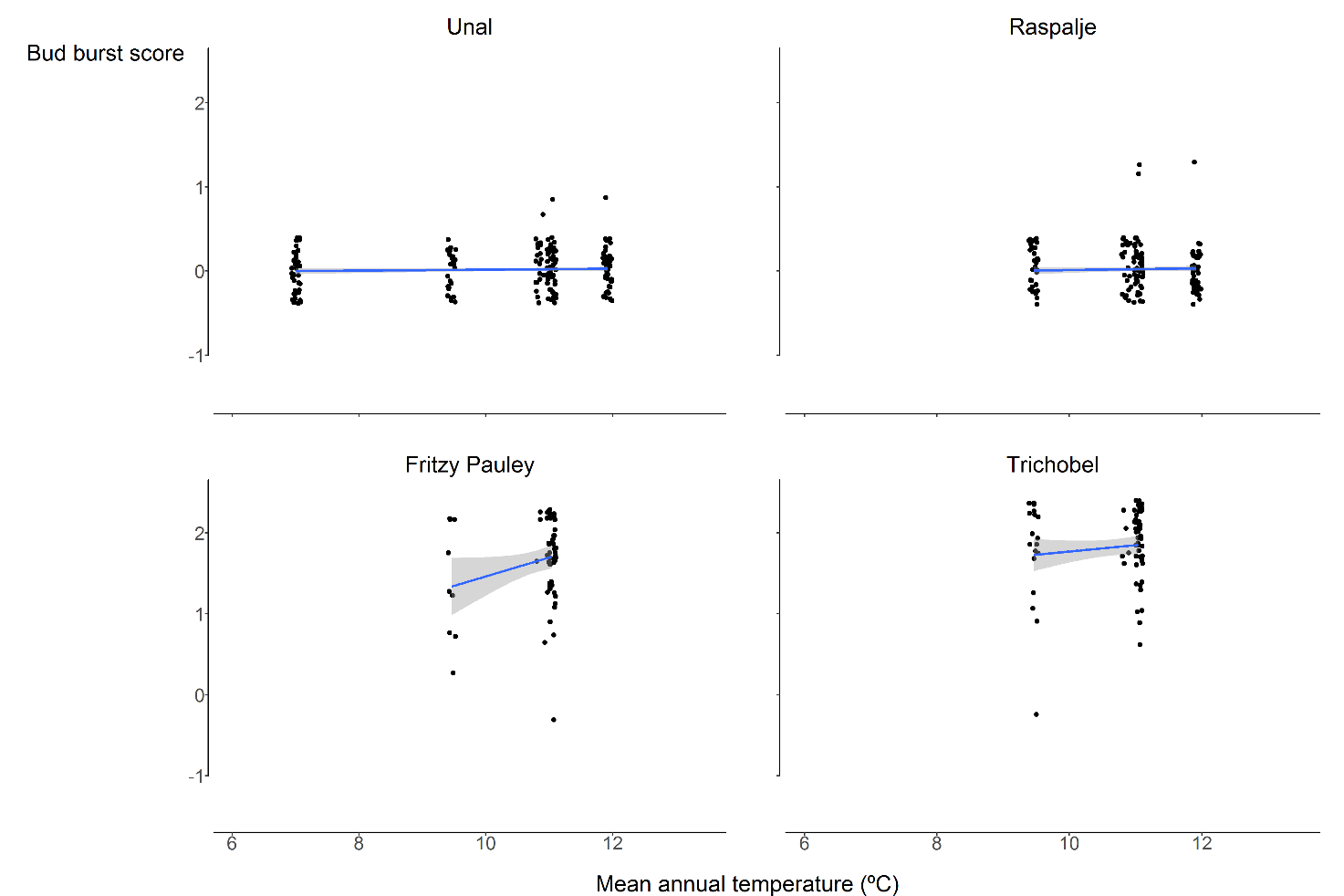
**

**S1 Fig. The relationship between mean annual temperature (MAT) and mean bud burst score in the cuttings of four different clones on 2^nd^ observation** (83th day of the year-DOY) where a, b, c and d represents respectively for genotypes Unal, Raspalje, Fritzy Pauley and Trichobel. On 83 DOY the variance in bud burst score for clone Beaupré was o (zero).
