# Supplementary material for: Transgenerational effects in asexually reproduced offspring of Populus
Source: PLoS One. 2018 Dec 6;13(12):e0208591. doi: 10.1371/journal.pone.0208591 (PMC6283561; doi:10.1371/journal.pone.0208591)
Supplement: S2 Fig — (DOCX) [file pone.0208591.s002.docx]

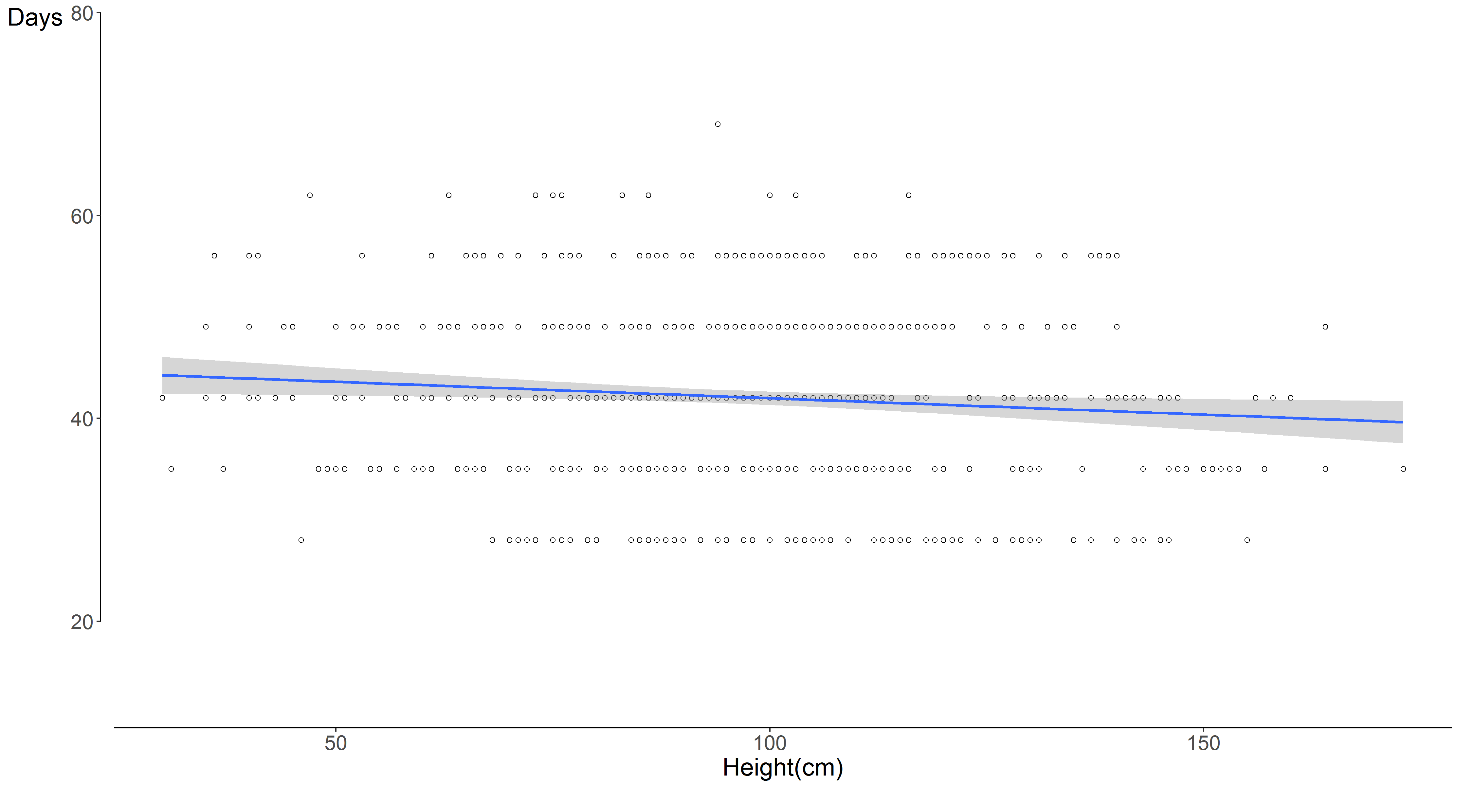


**S2 Fig. The relationship between number of days to bud burst in 2015 and the height of the seedlings during end of growing season in December 2014**
