# Supplementary material for: Transgenerational effects in asexually reproduced offspring of Populus
Source: PLoS One. 2018 Dec 6;13(12):e0208591. doi: 10.1371/journal.pone.0208591 (PMC6283561; doi:10.1371/journal.pone.0208591)
Supplement: S4 Fig — Mean weighted (bootstrapped) slopes of the relationship between the mean bud set score in 2014 and 2015 and day lengths on 1 May (a) and 1 January (b) experienced by the parent trees. Error bars denote 95% confidence interval (upper and lower) across the 500 bootstrapped values. Significances at the 95% level are denoted by *. “Earlier” means that buds set earlier with increasing temperature and “Later” means that buds set later with increasing temperature. (DOCX) [file pone.0208591.s004.docx]

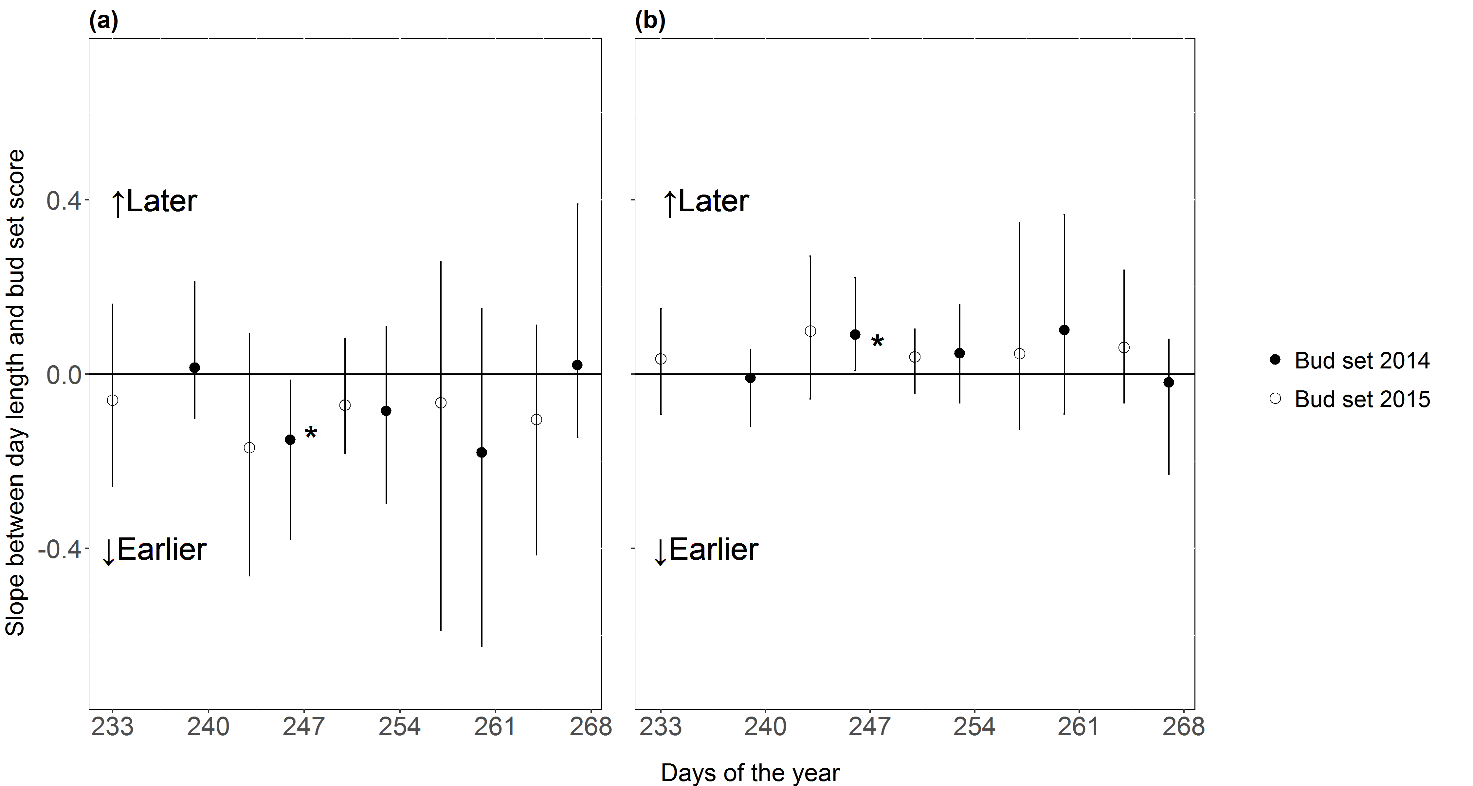


**S4** **Fig. Mean weighted (bootstrapped) slopes of the relationship between the mean bud set score in 2014 and 2015 and day lengths on 1 May (a) and 1 January (b) experienced by the parent trees**. Error bars denote 95% confidence interval (upper and lower) across the 500 bootstrapped values. Significances at the 95% level are denoted by *. “Earlier” means that buds set earlier with increasing temperature and “Later” means that buds set later with increasing temperature.
