# Supplementary material for: Transgenerational effects in asexually reproduced offspring of Populus
Source: PLoS One. 2018 Dec 6;13(12):e0208591. doi: 10.1371/journal.pone.0208591 (PMC6283561; doi:10.1371/journal.pone.0208591)
Supplement: S5 Fig — Error bars denote 95% confidence interval (upper and lower) across the 500 bootstrapped values. ‘Shorter’ means that growing season is shorter with increasing day length and ‘Longer’ means that growing season is longer with increasing day length. (DOCX) [file pone.0208591.s005.docx]

**
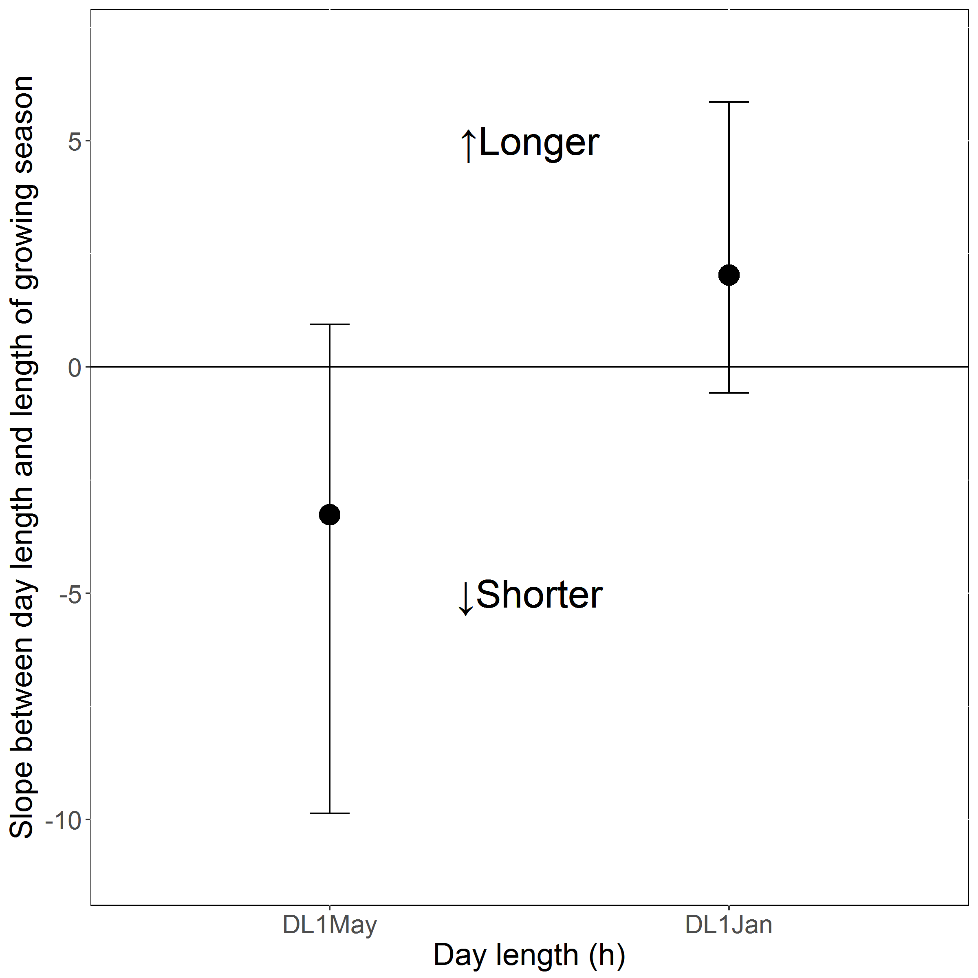
**

**S5 Fig. Mean weighted (bootstrapped) slopes of the relationship between the length of growing season in 2015 and day lengths on 1 May (DL1May) and 1 January (DL1Jan) experienced by the parent trees.** Error bars denote 95% confidence interval (upper and lower) across the 500 bootstrapped values. ‘Shorter’ means that growing season is shorter with increasing day length and ‘Longer’ means that growing season is longer with increasing day length.
