# Supplementary material for: Transgenerational effects in asexually reproduced offspring of Populus
Source: PLoS One. 2018 Dec 6;13(12):e0208591. doi: 10.1371/journal.pone.0208591 (PMC6283561; doi:10.1371/journal.pone.0208591)
Supplement: S6 Fig — Representation of Principal Coordinate Analysis (PCoA) for methylated (A) and non-methylated (B) loci. Samples are grouped per clone (countries are here BE = Belgium, FR = France, ES = Spain, IT = Italy and SE = Sweden). The first two coordinates (PCO1 and PCO2) are shown with the percentage of variance explained by them. Different point types represent individuals from different groups. (DOCX) [file pone.0208591.s006.docx]

**
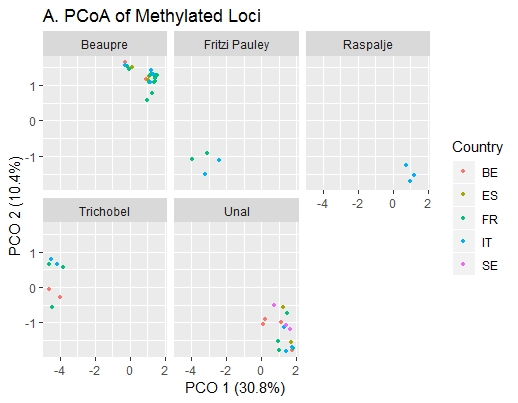

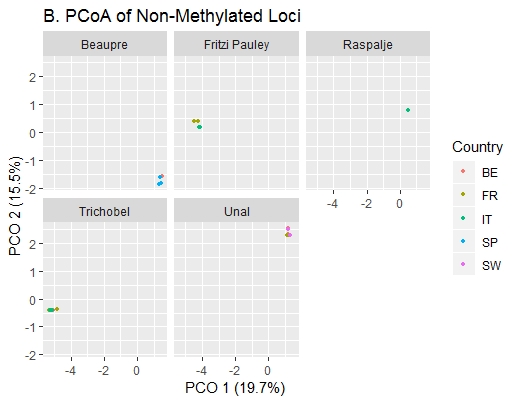
**

**S6 Fig. Representation of Principal Coordinate Analysis (PCoA) for methylated (A) and non-methylated (B) loci. Samples are grouped per clone (countries are here BE= Belgium, FR=France, ES=Spain, IT=Italy and SE=Sweden).** The first two coordinates (PCO1 and PCO2) are shown with the percentage of variance explained by them. Different point types represent individuals from different groups
