# Supplementary material for: Transgenerational effects in asexually reproduced offspring of Populus
Source: PLoS One. 2018 Dec 6;13(12):e0208591. doi: 10.1371/journal.pone.0208591 (PMC6283561; doi:10.1371/journal.pone.0208591)
Supplement: S1 Table — (DOCX) [file pone.0208591.s007.docx]

**S1** **Table.** **Description of the scoring systems of bud burst and bud set in poplar cuttings based on visual observations.**

| **Bud burst score** | **Description of visual evaluation** |
| --- | --- |
| 0 | Dormant bud; no sign of any physiological activity |
| 1 | Buds were slightly swollen and the bud scales reddishly coloured |
| 2 | Buds were fully swollen and turned towards a rounded shape, no sign of breakage of buds |
| 3 | Buds started breaking, wet and sticky, tip of reddish shoots appeared |
| 4 | Bud burst and reddish shoots turned towards a green colour, very young leaves could be observed |
| 5 | Green leaves started growing and venation of leaf could be observed |
| **Bud set score** |  |
| 3 | More than two rolled-up leaves |
| 2 | Last leaf (partially) rolled-up, other leaves fully stretched |
| 1 | Bud well visible, bud scales predominantly green colour, all leaves are stretched |
| 0 | Apical bud reddish-brown colour |
