# Supplementary material for: Transgenerational effects in asexually reproduced offspring of Populus
Source: PLoS One. 2018 Dec 6;13(12):e0208591. doi: 10.1371/journal.pone.0208591 (PMC6283561; doi:10.1371/journal.pone.0208591)
Supplement: S2 Table — (DOCX) [file pone.0208591.s008.docx]

**S2 Table. Number of individuals collected and monitored from respective genotpes and country for determination of DNA mathylation and bud phenology (bud burst and set).** In total, we samples 54 leaf samples for DNA methylation. But we removed one sample due to the mismatch after verifying the identity of the genotype.

| **Country** | **Name of the genotypes** | **No of individuals** | | | |
| --- | --- | --- | --- | --- | --- |
|  |  | **DNA methylation** | **Bud burst 2015** | **Bud set 2014** | **Bud set 2015** |
| Belgium |  |  |  |  |  |
|  | Beaupré | 2 | 18 | 19 | 18 |
|  | Fritzi Pauley |  | 4 | 4 | 3 |
|  | Raspalje |  | 20 | 20 | 19 |
|  | Trichobel | 2 | 5 | 5 | 5 |
|  | Unal | 3 | 20 | 20 | 19 |
| France (Beuxes) |  |  |  |  |  |
|  | Beaupré | 3 | 25 | 28 | 21 |
| France (Gueméne Penfao) | Beaupré | 4 | 49 | 48 | 50 |
|  | Fritzi Pauley | 2 | 50 | 47 | 48 |
|  | Raspalje |  | 49 | 48 | 49 |
|  | Trichobel | 4 | 49 | 42 | 49 |
|  | Unal | 4 | 50 | 50 | 48 |
| France (Saint-Usage) |  |  |  |  |  |
|  | Beaupré | 5 | 18 | 19 | 13 |
| Italy |  |  |  |  |  |
|  | Beaupré | 4 | 32 | 33 | 31 |
|  | Fritzi Pauley | 2 | 9 | 9 | 7 |
|  | Raspalje | 3 | 34 | 34 | 31 |
|  | Trichobel | 2 | 18 | 18 | 17 |
|  | Unal | 3 | 30 | 29 | 30 |
| Spain |  |  |  |  |  |
|  | Beaupré | 3 | 49 | 49 | 48 |
|  | Raspalje |  | 46 | 47 | 42 |
|  | Unal | 4 | 50 | 49 | 48 |
| Sweden | Unal | 3 | 39 | 38 | 38 |
