# Supplementary material for: Transgenerational effects in asexually reproduced offspring of Populus
Source: PLoS One. 2018 Dec 6;13(12):e0208591. doi: 10.1371/journal.pone.0208591 (PMC6283561; doi:10.1371/journal.pone.0208591)
Supplement: S3 Table — TA; annealing temperature, MP: multiplex. (DOCX) [file pone.0208591.s009.docx]

**S3** **Table.** **List of the microsatellite markers used for the genotype identification. TA; annealing temperature, MP: multiplex**

| **Sl. No.** | **Locus** | **LeftPrimer /forward (5′→3′)** | **RightPrimer/reverse (5′→3′)** | **Motif** | **Expected Product length** | **TA** | **MP** | **Dye** |
| --- | --- | --- | --- | --- | --- | --- | --- | --- |
| 1 | PMGC_14 | TTCAGAATGTGCATGATGG | GTGATGATCTCACCGTTTG | CTT | 179 - 227 | 52 | 1 | FAM |
| 2 | PMGC_2163 | CAATCGAAGGTAAGGTTAGTG | CGTTGGACATAGATCACACG | GA | 198 - 220 | 52 | 1 | NED |
| 3 | WPMS_05 | TTCTTTTTCAACTGCCTAACTT | TGATCCAATAACAGACAGAACA | GT | 263 - 291 | 52 | 1 | VIC |
| 4 | WPMS_16 | CTCGTACTATTTCCGATGATGACC | AGATTATTAGGTGGGCCAAGGACT | GTC | 128-167 | 52 | 1 | PET |
| 5 | ORPM 312 | GTGGGGATCAATCCAAAAGA | CCCATATCAAACCATTTGAAAAA | CCT | 189–201 |  | 2 | FAM |
| 6 | WPMS_20 | GTGCGCACATCTATGACTATCG | ATCTTGTAATTCTCCGGGCATCT | TTCTGG | 224 - 242 | 57 | 2 | NED |
| 7 | PTR2 | AAGAAGAACTCGAAGATGAAGAACT | ACTGACAAAACCCCTAATCTAACAA | TGG | 207 -228 | 57 | 2 | VIC |
| 8 | PTR7 | ATTTGATGCCTCTTCCTTCCAGT | TATTTTCATTTTCCCTTTGCTTT | (CT)5AT(CT) | 230 -250 | 57 | 2 | PET |
| 9 | WPMS_14 | CAGCCGCAGCCACTGAGAAATC | GCCTGCTGAGAAGACTGCCTTGAC | CGT | 221 - 304 | 57 | 3 | FAM |
| 10 | WPMS_15 | CAACAAACCATCAATGAAGAAGAC | AGAGGGTGTTGGGGGTGACTA | CCT | 188 - 203 | 57 | 3 | NED |
| 11 | WPMS_19 | AGCCACAGCAAATTCAGATGATGC | CCTGCTGAGAAGACTGCCTTGACA | CAG | 174-252 | 57 | 3 | VIC |
| 12 | WPMS_22 | ACATGCTACGTGTTTGGAATG | ATCGTATGGATGTAATTGTCTTA | TGA | 100-135 | 57 | 3 | FAM |
