# Supplementary material for: Transgenerational effects in asexually reproduced offspring of Populus
Source: PLoS One. 2018 Dec 6;13(12):e0208591. doi: 10.1371/journal.pone.0208591 (PMC6283561; doi:10.1371/journal.pone.0208591)
Supplement: S4 Table — (DOCX) [file pone.0208591.s010.docx]

**S4 Table.** **Primer combinations used, number of loci and estimated genotyping error rates**

| Primer combinations | Number of polymorphic fragments | Estimated genotyping error rate |
| --- | --- | --- |
| *Eco*RI + ACC / *Hpa*II-*Msp*I + TAC | 26 | 0.000 |
| *Eco*RI + ACC / *Hpa*II-*Msp*I + TAG | 38 | 0.025 |
| *Eco*RI + AGC / *Hpa*II-*Msp*I + TCC | 25 | 0.000 |
| *Eco*RI + AGC / *Hpa*II-*Msp*I + TCT | 33 | 0.029 |
| *Eco*RI + AGC / *Hpa*II-*Msp*I + TCG | 24 | 0.040 |
| *Eco*RI + AGC / *Hpa*II-*Msp*I + TAA | 61 | 0.024 |
| *Eco*RI + ACT / *Hpa*II-*Msp*I + TAG | 26 | 0.037 |
| *Total* | *233* |  |
| *Mean* |  | *0.022* |
