# Supplementary material for: Transgenerational effects in asexually reproduced offspring of Populus
Source: PLoS One. 2018 Dec 6;13(12):e0208591. doi: 10.1371/journal.pone.0208591 (PMC6283561; doi:10.1371/journal.pone.0208591)
Supplement: S5 Table — NA means no data available due to 0 (zero) variance in response variable. We used lmerTest package to extract the ρ values from the linear mixed effects models [66]. (PDF) [file pone.0208591.s011.pdf]

S5 Table. The results from the linear mixed effect models (in response to temperature variables and stem diameter). NA means no data available due to 0 (zero) variance in response variable. We used *lmerTest* package to extract the ρ values from the linear mixed effects models [1].

Fixed effects				MAT				MjulyT				MjanT			
Response	Clone	Observation	Variable	estimate	std. error	t value	p value	estimate	std. error	t value	p value	estimate	std. error	t value	p value
Bud set 2014	Beaupre	239	Temperature	0.01	0.10	0.12	0.91	0.04	0.09	0.45	0.65	0.00	0.07	-0.06	0.96
		239	Dia_stem	0.03	0.01	2.99	0.00	0.03	0.01	2.99	0.00	0.03	0.01	2.99	0.00
	Fritzy P.	239	Temperature	0.16	0.12	1.40	0.16	-0.03	0.34	-0.08	0.94	0.12	0.09	1.36	0.17
		239	Dia_stem	-0.02	0.03	-0.53	0.59	-0.01	0.03	-0.21	0.84	-0.01	0.03	-0.49	0.63
	Raspalje	239	Temperature	-0.04	0.04	-1.19	0.24	-0.03	0.02	-1.31	0.19	-0.02	0.03	-0.52	0.60
		239	Dia_stem	0.02	0.01	1.45	0.15	0.02	0.01	1.60	0.11	0.03	0.01	2.16	0.03
	Trichobel	239	Temperature	0.00	0.09	0.04	0.97	0.05	0.27	0.18	0.86	0.00	0.07	0.01	0.99
		239	Dia_stem	0.03	0.04	0.78	0.44	0.03	0.03	0.83	0.41	0.03	0.04	0.80	0.42
	Unal	239	Temperature	0.04	0.02	2.29	0.02	0.04	0.02	2.22	0.03	0.04	0.02	1.96	0.05
		239	Dia_stem	0.01	0.01	1.18	0.24	0.01	0.01	0.87	0.38	0.02	0.01	1.42	0.16
	Beaupre	246	Temperature	-0.03	0.09	-0.34	0.73	0.00	0.08	-0.04	0.97	-0.02	0.06	-0.30	0.76
		246	Dia_stem	0.01	0.01	0.48	0.63	0.01	0.01	0.43	0.67	0.01	0.01	0.48	0.63
	Fritzy P.	246	Temperature	-0.18	0.16	-1.07	0.28	0.13	0.41	0.32	0.75	-0.14	0.12	-1.14	0.26
		246	Dia_stem	0.02	0.04	0.39	0.70	0.00	0.04	-0.05	0.96	0.02	0.04	0.40	0.69
	Raspalje	246	Temperature	0.02	0.06	0.36	0.72	0.04	0.03	1.23	0.22	-0.01	0.05	-0.26	0.79
		246	Dia_stem	0.04	0.02	1.92	0.05	0.04	0.02	2.37	0.02	0.03	0.02	1.79	0.07
	Trichobel	246	Temperature	-0.10	0.12	-0.86	0.39	-0.24	0.35	-0.70	0.48	-0.06	0.09	-0.71	0.48
		246	Dia_stem	-0.01	0.05	-0.16	0.87	-0.02	0.05	-0.44	0.66	-0.01	0.05	-0.25	0.80
	Unal	246	Temperature	0.04	0.02	1.92	0.05	0.04	0.03	1.35	0.18	0.03	0.02	1.84	0.07
		246	Dia_stem	0.00	0.01	0.38	0.71	0.00	0.01	-0.13	0.90	0.01	0.01	0.72	0.47
	Beaupre	253	Temperature	0.02	0.08	0.22	0.83	0.01	0.07	0.15	0.88	0.01	0.06	0.24	0.81
		253	Dia_stem	0.01	0.01	0.36	0.72	0.01	0.01	0.35	0.73	0.01	0.01	0.37	0.71
	Fritzy P.	253	Temperature	-0.07	0.17	-0.43	0.67	0.04	0.39	0.10	0.92	-0.06	0.12	-0.45	0.65
		253	Dia_stem	0.00	0.04	0.08	0.93	-0.01	0.04	-0.14	0.89	0.00	0.04	0.08	0.93
	Raspalje	253	Temperature	0.02	0.05	0.41	0.68	-0.01	0.03	-0.31	0.76	0.04	0.04	0.93	0.35
		253	Dia_stem	0.01	0.01	0.93	0.35	0.01	0.01	0.66	0.51	0.01	0.01	1.10	0.27
	Trichobel	253	Temperature	-0.15	0.13	-1.19	0.24	-0.05	0.39	-0.13	0.90	-0.10	0.09	-1.13	0.26

		253	Dia_stem	-0.02	0.05	-0.46	0.64	-0.04	0.05	-0.81	0.42	-0.03	0.05	-0.52	0.60
	Unal	253	Temperature	0.06	0.02	2.79	0.01	0.06	0.03	1.87	0.06	0.06	0.02	2.66	0.01
		253	Dia_stem	0.01	0.01	0.62	0.54	0.00	0.02	0.03	0.97	0.01	0.01	1.05	0.29
	Beaupre	260	Temperature	-0.03	0.04	-0.78	0.44	-0.06	0.02	-2.36	0.02	0.00	0.03	0.03	0.98
		260	Dia_stem	-0.01	0.01	-0.91	0.37	-0.01	0.01	-0.52	0.60	-0.01	0.01	-1.01	0.31
	Fritzy P.	260	Temperature	-0.23	0.17	-1.31	0.19	0.37	0.51	0.73	0.47	-0.19	0.13	-1.47	0.14
		260	Dia_stem	0.02	0.05	0.40	0.69	0.00	0.04	0.04	0.97	0.02	0.04	0.45	0.65
	Raspalje	260	Temperature	0.05	0.07	0.73	0.46	0.06	0.03	2.00	0.05	-0.01	0.07	-0.20	0.84
		260	Dia_stem	0.04	0.02	2.05	0.04	0.05	0.02	2.53	0.01	0.04	0.02	1.81	0.07
	Trichobel	260	Temperature	-0.27	0.09	-2.94	0.00	0.02	0.52	0.05	0.96	-0.19	0.07	-2.79	0.01
		260	Dia_stem	0.02	0.04	0.58	0.56	0.02	0.04	0.50	0.62	0.02	0.04	0.45	0.66
	Unal	260	Temperature	0.04	0.03	1.31	0.19	0.06	0.02	3.16	0.00	0.02	0.03	0.83	0.40
		260	Dia_stem	-0.01	0.01	-0.96	0.34	-0.02	0.01	-1.60	0.11	-0.01	0.01	-0.83	0.41
	Beaupre	267	Temperature	-0.03	0.03	-1.07	0.28	0.01	0.02	0.33	0.74	-0.03	0.02	-1.77	0.08
		267	Dia_stem	0.00	0.01	-0.27	0.78	0.00	0.01	-0.40	0.69	0.00	0.01	-0.20	0.84
	Fritzy P.	267	Temperature	0.06	0.37	0.16	0.87	-0.74	0.22	-3.36	0.00	0.10	0.23	0.44	0.66
		267	Dia_stem	0.02	0.02	0.79	0.43	0.01	0.02	0.64	0.52	0.02	0.02	0.76	0.45
	Raspalje	267	Temperature	0.04	0.04	0.99	0.32	0.01	0.02	0.59	0.55	0.02	0.03	0.70	0.49
		267	Dia_stem	0.01	0.01	0.67	0.50	0.01	0.01	0.40	0.69	0.00	0.01	0.39	0.70
	Trichobel	267	Temperature	-0.09	0.07	-1.30	0.20	-0.06	0.24	-0.24	0.81	-0.06	0.05	-1.20	0.23
		267	Dia_stem	0.00	0.03	-0.13	0.90	-0.01	0.03	-0.40	0.69	-0.01	0.03	-0.20	0.84
	Unal	267	Temperature	0.01	0.02	0.44	0.66	0.02	0.02	1.12	0.26	0.00	0.02	0.05	0.96
		267	Dia_stem	-0.02	0.01	-1.68	0.09	-0.02	0.01	-1.88	0.06	-0.02	0.01	-1.63	0.10
Bud set 2015	Beaupre	233	Temperature	0.08	0.13	0.62	0.53	0.10	0.10	0.95	0.34	0.03	0.10	0.33	0.74
		233	Dia_stem	0.06	0.02	3.06	0.00	0.06	0.02	3.12	0.00	0.06	0.02	3.03	0.00
	Fritzy P.	233	Temperature	-0.02	0.12	-0.15	0.88	-0.23	0.31	-0.73	0.46	0.00	0.09	0.02	0.99
		233	Dia_stem	-0.02	0.03	-0.51	0.61	-0.01	0.03	-0.49	0.62	-0.02	0.03	-0.57	0.57
	Raspalje	233	Temperature	-0.04	0.08	-0.50	0.62	-0.06	0.03	-1.78	0.08	0.01	0.08	0.15	0.88
		233	Dia_stem	0.01	0.02	0.52	0.60	0.01	0.02	0.30	0.76	0.01	0.02	0.77	0.44
	Trichobel	233	Temperature	0.03	0.14	0.22	0.83	0.33	0.24	1.38	0.17	0.00	0.10	-0.04	0.97
		233	Dia_stem	0.02	0.03	0.52	0.61	0.02	0.03	0.69	0.49	0.02	0.03	0.56	0.58
	Unal	233	Temperature	0.03	0.03	0.85	0.39	0.04	0.03	1.16	0.24	0.02	0.03	0.66	0.51
		233	Dia_stem	0.03	0.02	1.37	0.17	0.02	0.02	1.00	0.32	0.03	0.02	1.60	0.11

Beaupre	243	Temperature	-0.10	0.14	-0.69	0.49	0.02	0.13	0.13	0.90	-0.09	0.10	-0.92	0.36
	243	Dia_stem	0.06	0.02	2.75	0.01	0.06	0.02	2.59	0.01	0.07	0.02	2.85	0.00
Fritzy P.	243	Temperature	-0.04	0.15	-0.30	0.77	0.01	0.37	0.03	0.98	-0.03	0.11	-0.30	0.76
	243	Dia_stem	0.03	0.04	0.74	0.46	0.02	0.04	0.67	0.50	0.03	0.04	0.74	0.46
Raspalje	243	Temperature	-0.12	0.08	-1.53	0.13	-0.08	0.04	-1.94	0.05	-0.05	0.09	-0.58	0.56
	243	Dia_stem	0.00	0.03	0.05	0.96	0.00	0.02	0.17	0.86	0.01	0.03	0.35	0.73
Trichobel	243	Temperature	0.05	0.40	0.13	0.89	0.79	0.40	1.96	0.05	-0.03	0.27	-0.12	0.90
	243	Dia_stem	-0.03	0.05	-0.67	0.50	-0.02	0.05	-0.46	0.64	-0.03	0.05	-0.65	0.52
Unal	243	Temperature	0.08	0.05	1.58	0.11	0.12	0.04	2.82	0.00	0.05	0.06	0.90	0.37
	243	Dia_stem	0.01	0.03	0.20	0.84	-0.01	0.03	-0.42	0.67	0.01	0.03	0.36	0.72
Beaupre	250	Temperature	-0.02	0.09	-0.26	0.79	0.03	0.08	0.39	0.70	-0.03	0.06	-0.54	0.59
	250	Dia_stem	0.07	0.02	3.18	0.00	0.06	0.02	3.01	0.00	0.07	0.02	3.41	0.00
Fritzy P.	250	Temperature	-0.19	0.19	-1.04	0.30	-0.41	0.47	-0.86	0.39	-0.12	0.14	-0.85	0.40
	250	Dia_stem	0.02	0.05	0.41	0.68	0.01	0.04	0.16	0.87	0.01	0.05	0.32	0.75
Raspalje	250	Temperature	-0.06	0.12	-0.47	0.64	-0.06	0.06	-0.94	0.35	-0.01	0.12	-0.12	0.90
	250	Dia_stem	0.03	0.03	0.96	0.34	0.03	0.03	0.95	0.34	0.03	0.03	1.14	0.25
Trichobel	250	Temperature	0.06	0.13	0.47	0.64	0.29	0.38	0.77	0.44	0.03	0.10	0.31	0.76
	250	Dia_stem	-0.01	0.05	-0.12	0.91	0.00	0.05	-0.06	0.95	0.00	0.05	-0.05	0.96
Unal	250	Temperature	0.06	0.06	0.91	0.37	0.11	0.04	2.75	0.01	0.03	0.06	0.49	0.62
	250	Dia_stem	0.03	0.03	1.24	0.21	0.02	0.02	0.61	0.54	0.04	0.03	1.36	0.17
Beaupre	257	Temperature	-0.04	0.04	-0.98	0.33	0.01	0.03	0.22	0.83	-0.05	0.03	-1.50	0.13
	257	Dia_stem	0.03	0.01	2.63	0.01	0.04	0.01	2.68	0.01	0.03	0.01	2.65	0.01
Fritzy P.	257	Temperature	-0.22	0.20	-1.12	0.26	0.48	0.57	0.84	0.40	-0.19	0.15	-1.31	0.19
	257	Dia_stem	0.01	0.05	0.10	0.92	-0.01	0.05	-0.29	0.77	0.01	0.05	0.13	0.90
Raspalje	257	Temperature	-0.04	0.10	-0.38	0.70	-0.07	0.05	-1.52	0.13	0.04	0.09	0.41	0.68
	257	Dia_stem	0.03	0.03	1.02	0.31	0.02	0.03	0.73	0.47	0.04	0.03	1.49	0.14
Trichobel	257	Temperature	0.05	0.15	0.34	0.73	0.30	0.42	0.73	0.47	0.02	0.11	0.20	0.84
	257	Dia_stem	-0.02	0.05	-0.28	0.78	-0.01	0.05	-0.27	0.78	-0.01	0.05	-0.23	0.82
Unal	257	Temperature	-0.02	0.04	-0.40	0.69	0.01	0.04	0.12	0.90	-0.02	0.04	-0.54	0.59
	257	Dia_stem	0.04	0.02	2.09	0.04	0.04	0.02	1.81	0.07	0.04	0.02	2.12	0.03
Beaupre	264	Temperature	-0.03	0.02	-1.44	0.15	0.00	0.02	-0.25	0.80	-0.02	0.01	-1.69	0.09
	264	Dia_stem	0.01	0.01	1.87	0.06	0.01	0.01	1.91	0.06	0.01	0.01	1.87	0.06
Fritzy P.	264	Temperature	-0.03	0.16	-0.22	0.83	0.26	0.41	0.63	0.53	-0.04	0.12	-0.36	0.72

		264	Dia_stem	-0.05	0.04	-1.22	0.22	-0.06	0.04	-1.47	0.14	-0.05	0.04	-1.19	0.23
	Raspalje	264	Temperature	-0.06	0.07	-0.85	0.40	-0.04	0.04	-1.16	0.24	-0.01	0.06	-0.11	0.91
		264	Dia_stem	0.02	0.02	0.69	0.49	0.02	0.02	0.71	0.48	0.03	0.02	1.29	0.20
	Trichobel	264	Temperature	-0.04	0.28	-0.13	0.90	0.60	0.36	1.69	0.09	-0.07	0.18	-0.38	0.71
		264	Dia_stem	0.02	0.05	0.38	0.70	0.02	0.04	0.40	0.69	0.02	0.05	0.42	0.67
	Unal	264	Temperature	0.01	0.03	0.33	0.74	0.01	0.03	0.34	0.74	0.01	0.03	0.38	0.70
		264	Dia_stem	0.00	0.01	0.34	0.73	0.00	0.01	0.24	0.81	0.01	0.01	0.39	0.69
Budburst 2015	Beaupre	83	Temperature	NA	NA	NA	NA	NA	NA	NA	NA	NA	NA	NA	NA
		83	Dia_stem	NA	NA	NA	NA	NA	NA	NA	NA	NA	NA	NA	NA
	Fritzy P.	83	Temperature	-0.25	0.15	-1.71	0.09	-0.12	0.06	-1.85	0.07	0.28	0.16	1.82	0.07
		83	Dia_stem	-0.01	0.03	-0.18	0.86	-0.01	0.04	-0.31	0.75	-0.01	0.04	-0.39	0.70
	Raspalje	83	Temperature	-0.01	0.02	-0.28	0.80	0.00	0.01	-0.43	0.72	0.03	0.02	1.32	0.19
		83	Dia_stem	0.00	0.00	0.07	0.94	0.00	0.00	0.03	0.98	0.00	0.01	0.57	0.57
	Trichobel	83	Temperature	-0.13	0.09	-1.46	0.15	-0.05	0.04	-1.42	0.16	0.10	0.09	1.11	0.27
		83	Dia_stem	-0.02	0.03	-0.57	0.57	-0.02	0.03	-0.61	0.54	-0.02	0.03	-0.58	0.56
	Unal	83	Temperature	0.00	0.01	0.77	0.45	0.00	0.01	-0.14	0.89	0.00	0.00	1.16	0.25
		83	Dia_stem	0.00	0.00	-0.55	0.59	0.00	0.01	0.07	0.94	0.00	0.00	-0.60	0.55
	Beaupre	90	Temperature	0.00	0.01	0.27	0.79	0.00	0.00	0.83	0.40	0.00	0.01	0.20	0.85
		90	Dia_stem	0.00	0.00	0.62	0.54	0.00	0.00	0.55	0.58	0.00	0.00	0.73	0.47
	Fritzy P.	90	Temperature	-0.09	0.12	-0.79	0.44	-0.05	0.05	-0.92	0.36	0.14	0.13	1.10	0.28
		90	Dia_stem	0.06	0.03	2.11	0.04	0.06	0.03	1.97	0.05	0.05	0.03	1.81	0.08
	Raspalje	90	Temperature	-0.08	0.10	-0.76	0.53	-0.04	0.04	-0.89	0.48	-0.10	0.15	-0.64	0.59
		90	Dia_stem	0.00	0.02	-0.04	0.97	0.00	0.02	-0.06	0.95	-0.01	0.02	-0.31	0.76
	Trichobel	90	Temperature	-0.05	0.14	-0.34	0.78	-0.03	0.06	-0.58	0.67	0.16	0.10	1.66	0.10
		90	Dia_stem	0.06	0.03	1.70	0.09	0.06	0.03	1.69	0.10	0.05	0.03	1.57	0.12
	Unal	90	Temperature	0.02	0.02	0.84	0.45	0.02	0.03	0.66	0.53	0.01	0.01	1.00	0.32
		90	Dia_stem	-0.01	0.02	-0.64	0.52	-0.01	0.02	-0.59	0.55	-0.01	0.01	-0.58	0.56
	Beaupre	97	Temperature	0.03	0.03	1.11	0.27	0.00	0.01	0.21	0.85	0.04	0.02	1.72	0.09
		97	Dia_stem	0.00	0.01	0.42	0.68	0.00	0.01	0.81	0.43	0.00	0.00	0.74	0.46
	Fritzy P.	97	Temperature	0.06	0.20	0.29	0.81	0.01	0.09	0.13	0.92	0.13	0.26	0.52	0.71
		97	Dia_stem	0.00	0.02	0.14	0.89	0.00	0.02	0.11	0.91	0.00	0.02	0.02	0.98
	Raspalje	97	Temperature	-0.08	0.08	-0.90	0.37	-0.03	0.03	-0.85	0.40	-0.06	0.12	-0.50	0.62
		97	Dia_stem	0.03	0.02	1.45	0.15	0.03	0.02	1.35	0.18	0.03	0.03	1.04	0.30

Trichobel	97	Temperature	0.02	0.13	0.13	0.91	0.00	0.06	-0.03	0.98	0.09	0.12	0.80	0.76
	97	Dia_stem	-0.02	0.03	-0.57	0.57	-0.02	0.03	-0.59	0.56	-0.02	0.03	-0.66	0.51
Unal	97	Temperature	0.03	0.02	1.27	0.21	0.02	0.03	0.59	0.57	0.02	0.01	1.14	0.25
	97	Dia_stem	-0.02	0.02	-0.93	0.36	-0.01	0.02	-0.55	0.58	-0.01	0.02	-0.65	0.52
Beaupre	104	Temperature	0.05	0.10	0.55	0.59	0.03	0.04	0.81	0.42	0.00	0.08	0.01	0.99
	104	Dia_stem	-0.01	0.02	-0.69	0.49	-0.01	0.02	-0.68	0.50	-0.01	0.02	-0.56	0.57
Fritzy P.	104	Temperature	0.00	0.15	0.03	0.98	0.00	0.06	0.00	1.00	0.01	0.16	0.06	0.95
	104	Dia_stem	0.09	0.03	2.46	0.02	0.09	0.04	2.40	0.02	0.08	0.04	2.32	0.02
Raspalje	104	Temperature	-0.14	0.12	-1.18	0.24	-0.05	0.05	-1.09	0.28	0.07	0.16	0.45	0.65
	104	Dia_stem	-0.01	0.03	-0.45	0.65	-0.02	0.03	-0.58	0.56	-0.01	0.04	-0.30	0.76
Trichobel	104	Temperature	0.01	0.22	0.04	0.97	-0.01	0.10	-0.12	0.92	0.20	0.20	0.97	0.67
	104	Dia_stem	-0.03	0.04	-0.74	0.46	-0.03	0.04	-0.75	0.45	-0.03	0.04	-0.80	0.43
Unal	104	Temperature	0.03	0.05	0.53	0.62	0.00	0.06	-0.07	0.94	0.02	0.03	0.71	0.52
	104	Dia_stem	-0.04	0.03	-1.13	0.26	-0.03	0.04	-0.74	0.46	-0.04	0.03	-1.20	0.24
Beaupre	111	Temperature	0.17	0.17	1.01	0.31	0.09	0.07	1.30	0.20	-0.02	0.14	-0.17	0.86
	111	Dia_stem	-0.05	0.03	-1.40	0.16	-0.04	0.03	-1.35	0.18	-0.04	0.03	-1.16	0.25
Fritzy P.	111	Temperature	NA	NA	NA	NA	NA	NA	NA	NA	NA	NA	NA	NA
	111	Dia_stem	NA	NA	NA	NA	NA	NA	NA	NA	NA	NA	NA	NA
Raspalje	111	Temperature	-0.14	0.14	-0.97	0.33	-0.05	0.06	-0.89	0.37	0.22	0.19	1.13	0.26
	111	Dia_stem	-0.06	0.04	-1.58	0.12	-0.07	0.04	-1.69	0.09	-0.04	0.04	-1.02	0.31
Trichobel	111	Temperature	0.00	0.02	0.07	0.94	0.00	0.01	0.12	0.91	-0.01	0.02	-0.21	0.83
	111	Dia_stem	-0.01	0.01	-1.18	0.24	-0.01	0.01	-1.15	0.25	-0.01	0.01	-1.08	0.28
Unal	111	Temperature	0.02	0.08	0.24	0.82	-0.01	0.09	-0.15	0.89	0.03	0.06	0.44	0.69
	111	Dia_stem	-0.05	0.04	-1.15	0.25	-0.04	0.04	-0.92	0.36	-0.05	0.04	-1.23	0.22
Beaupre	118	Temperature	0.07	0.16	0.47	0.67	0.08	0.06	1.26	0.21	-0.17	0.13	-1.31	0.19
	118	Dia_stem	-0.02	0.03	-0.78	0.44	-0.03	0.03	-0.86	0.39	-0.02	0.03	-0.66	0.51
Fritzy P.	118	Temperature	NA	NA	NA	NA	NA	NA	NA	NA	NA	NA	NA	NA
	118	Dia_stem	NA	NA	NA	NA	NA	NA	NA	NA	NA	NA	NA	NA
Raspalje	118	Temperature	-0.06	0.07	-0.77	0.52	-0.02	0.03	-0.69	0.57	0.17	0.07	2.30	0.02
	118	Dia_stem	-0.03	0.02	-1.49	0.15	-0.03	0.02	-1.50	0.14	-0.02	0.02	-1.12	0.27
Trichobel	118	Temperature	0.03	0.07	0.42	0.68	0.02	0.03	0.52	0.61	-0.05	0.08	-0.70	0.48
	118	Dia_stem	0.01	0.02	0.20	0.84	0.01	0.03	0.25	0.81	0.01	0.03	0.34	0.73
Unal	118	Temperature	0.01	0.03	0.34	0.75	-0.01	0.04	-0.36	0.72	0.02	0.02	0.84	0.46

	118	Dia_stem	-0.03	0.02	-1.23	0.22	-0.02	0.03	-0.72	0.47	-0.03	0.02	-1.52	0.14
Beaupre	125	Temperature	-0.01	0.11	-0.05	0.97	0.04	0.05	0.85	0.46	-0.12	0.09	-1.42	0.16
	125	Dia_stem	-0.01	0.02	-0.63	0.54	-0.02	0.02	-0.79	0.44	-0.01	0.02	-0.63	0.53
Fritzy P.	125	Temperature	NA	NA	NA	NA	NA	NA	NA	NA	NA	NA	NA	NA
	125	Dia_stem	NA	NA	NA	NA	NA	NA	NA	NA	NA	NA	NA	NA
Raspalje	125	Temperature	-0.01	0.01	-1.01	0.32	0.00	0.00	-1.24	0.22	0.00	0.02	0.19	0.87
	125	Dia_stem	-0.01	0.00	-3.64	0.00	-0.01	0.00	-3.78	0.00	-0.01	0.00	-3.33	0.00
Trichobel	125	Temperature	NA	NA	NA	NA	NA	NA	NA	NA	NA	NA	NA	NA
	125	Dia_stem	NA	NA	NA	NA	NA	NA	NA	NA	NA	NA	NA	NA
Unal	125	Temperature	0.00	0.01	0.15	0.89	0.00	0.01	-0.17	0.87	0.00	0.01	0.62	0.58
	125	Dia_stem	-0.01	0.01	-0.65	0.52	0.00	0.01	-0.41	0.68	-0.01	0.01	-0.90	0.38

Reference

1. Kuznetsova A, Brockhoff PB, Christensen RHB. “lmerTest Package: Tests in Linear Mixed Effects Models.”. Journal of Statistical Software. 2017;82(13):1-26. doi: doi: 10.18637/jss.v082.i13
