# Supplementary material for: Transgenerational effects in asexually reproduced offspring of Populus
Source: PLoS One. 2018 Dec 6;13(12):e0208591. doi: 10.1371/journal.pone.0208591 (PMC6283561; doi:10.1371/journal.pone.0208591)
Supplement: S6 Table — NA means no data available due to 0 (zero) variance in response variable. (PDF) [file pone.0208591.s012.pdf]

S6 Table. The results from the linear mixed effect models (in response to day lengths and stem diameter). NA means no data available due to 0 (zero) variance in response variable. We used *lmerTest* package to extract the ρ values from the linear mixed effects models [1].

Fixed effects											
				Day length1 May (DLMay)				Day length 1 January (DLJan)			
Response	Clone	Observation	Variable	estimate	std. error	t value	p value	estimate	std. error	t value	p value
Bud set 2014	Beaupre	239	Day length	-0.06	0.47	-0.14	0.89	0.04	0.28	0.13	0.90
		239	Dia_stem	0.03	0.01	2.99	0.00	0.03	0.01	2.99	0.00
	Fritzy P.	239	Day length	0.53	0.58	0.91	0.36	-0.31	0.34	-0.90	0.37
		239	Dia_stem	-0.01	0.03	-0.18	0.86	-0.01	0.03	-0.18	0.86
	Raspalje	239	Day length	0.10	0.10	0.94	0.35	-0.06	0.06	-0.93	0.35
		239	Dia_stem	0.02	0.01	2.18	0.03	0.02	0.01	2.19	0.03
	Trichobel	239	Day length	-0.04	0.49	-0.08	0.94	0.02	0.29	0.08	0.94
		239	Dia_stem	0.03	0.04	0.86	0.39	0.03	0.04	0.86	0.39
	Unal	239	Day length	-0.13	0.05	-2.59	0.01	0.07	0.03	2.58	0.01
		239	Dia_stem	0.00	0.01	0.24	0.81	0.00	0.01	0.27	0.79
	Beaupre	246	Day length	0.03	0.44	0.06	0.95	-0.02	0.26	-0.06	0.95
		246	Dia_stem	0.01	0.01	0.44	0.66	0.01	0.01	0.44	0.66
	Fritzy P.	246	Day length	-0.81	0.80	-1.00	0.32	0.47	0.47	1.00	0.32
		246	Dia_stem	0.01	0.04	0.19	0.85	0.01	0.04	0.18	0.86
	Raspalje	246	Day length	-0.18	0.15	-1.18	0.24	0.10	0.09	1.17	0.24
		246	Dia_stem	0.04	0.02	2.31	0.02	0.04	0.02	2.30	0.02
	Trichobel	246	Day length	-0.11	0.66	-0.17	0.87	0.06	0.39	0.16	0.87
		246	Dia_stem	-0.02	0.05	-0.43	0.67	-0.02	0.05	-0.43	0.67
	Unal	246	Day length	-0.13	0.06	-2.09	0.04	0.07	0.03	2.12	0.03
		246	Dia_stem	-0.01	0.01	-0.41	0.68	-0.01	0.01	-0.40	0.69
	Beaupre	253	Day length	0.11	0.39	0.28	0.78	-0.07	0.23	-0.29	0.77
		253	Dia_stem	0.01	0.01	0.40	0.69	0.01	0.01	0.41	0.68
	Fritzy P.	253	Day length	-0.32	0.82	-0.39	0.70	0.19	0.48	0.39	0.70
		253	Dia_stem	0.00	0.04	-0.01	1.00	0.00	0.04	-0.01	0.99
	Raspalje	253	Day length	0.11	0.14	0.77	0.44	-0.07	0.08	-0.79	0.43
		253	Dia_stem	0.01	0.01	0.61	0.54	0.01	0.01	0.60	0.55
	Trichobel	253	Day length	-0.53	0.66	-0.79	0.43	0.31	0.39	0.79	0.43

		253	Dia_stem	-0.04	0.05	-0.79	0.43	-0.04	0.05	-0.79	0.43
	Unal	253	Day length	-0.22	0.07	-3.16	0.00	0.12	0.04	3.17	0.00
		253	Dia_stem	-0.01	0.02	-0.57	0.57	-0.01	0.02	-0.55	0.58
	Beaupre	260	Day length	0.33	0.14	2.35	0.02	-0.20	0.08	-2.35	0.02
		260	Dia_stem	-0.01	0.01	-0.49	0.62	-0.01	0.01	-0.49	0.62
	Fritzy P.	260	Day length	-1.27	0.85	-1.49	0.14	0.74	0.50	1.48	0.14
		260	Dia_stem	0.01	0.04	0.23	0.82	0.01	0.04	0.22	0.83
	Raspalje	260	Day length	-0.31	0.16	-1.96	0.05	0.18	0.09	1.95	0.05
		260	Dia_stem	0.04	0.02	2.32	0.02	0.04	0.02	2.31	0.02
	Trichobel	260	Day length	-0.63	0.83	-0.75	0.45	0.36	0.49	0.75	0.46
		260	Dia_stem	0.02	0.04	0.47	0.64	0.02	0.04	0.47	0.64
	Unal	260	Day length	-0.12	0.07	-1.61	0.11	0.06	0.04	1.54	0.12
		260	Dia_stem	-0.02	0.01	-1.31	0.19	-0.02	0.01	-1.28	0.20
	Beaupre	267	Day length	-0.08	0.13	-0.62	0.53	0.05	0.08	0.62	0.54
		267	Dia_stem	0.00	0.01	-0.37	0.71	0.00	0.01	-0.37	0.71
	Fritzy P.	267	Day length	1.06	0.92	1.15	0.25	-0.62	0.53	-1.16	0.24
		267	Dia_stem	0.02	0.02	0.67	0.50	0.02	0.02	0.67	0.50
	Raspalje	267	Day length	-0.04	0.10	-0.35	0.73	0.02	0.06	0.35	0.73
		267	Dia_stem	0.00	0.01	0.22	0.83	0.00	0.01	0.22	0.83
	Trichobel	267	Day length	-0.27	0.40	-0.66	0.51	0.15	0.24	0.65	0.52
		267	Dia_stem	-0.01	0.03	-0.42	0.68	-0.01	0.03	-0.42	0.68
	Unal	267	Day length	-0.04	0.05	-0.82	0.41	0.02	0.03	0.79	0.43
		267	Dia_stem	-0.02	0.01	-1.77	0.08	-0.02	0.01	-1.76	0.08
Bud set 2015	Beaupre	233	Day length	-0.31	0.60	-0.52	0.60	0.18	0.35	0.51	0.61
		233	Dia_stem	0.06	0.02	3.05	0.00	0.06	0.02	3.05	0.00
	Fritzy P.	233	Day length	0.26	0.63	0.42	0.68	-0.16	0.37	-0.42	0.67
		233	Dia_stem	-0.02	0.03	-0.67	0.50	-0.02	0.03	-0.67	0.50
	Raspalje	233	Day length	0.27	0.17	1.58	0.11	-0.16	0.10	-1.56	0.12
		233	Dia_stem	0.01	0.02	0.70	0.49	0.01	0.02	0.70	0.48
	Trichobel	233	Day length	-0.28	0.56	-0.51	0.61	0.17	0.33	0.52	0.61
		233	Dia_stem	0.02	0.03	0.66	0.51	0.02	0.03	0.66	0.51
	Unal	233	Day length	-0.08	0.10	-0.78	0.44	0.04	0.06	0.75	0.45
		233	Dia_stem	0.02	0.02	0.93	0.35	0.02	0.02	0.96	0.34

Beaupre	243	Day length	-0.22	0.70	-0.32	0.75	0.13	0.42	0.32	0.75
	243	Dia_stem	0.06	0.02	2.60	0.01	0.06	0.02	2.60	0.01
Fritzy P.	243	Day length	-0.19	0.76	-0.25	0.80	0.11	0.45	0.25	0.80
	243	Dia_stem	0.03	0.04	0.71	0.48	0.03	0.04	0.71	0.48
Raspalje	243	Day length	0.30	0.21	1.46	0.15	-0.18	0.12	-1.44	0.15
	243	Dia_stem	0.02	0.02	0.78	0.44	0.02	0.02	0.79	0.43
Trichobel	243	Day length	-0.83	1.30	-0.64	0.53	0.49	0.76	0.64	0.52
	243	Dia_stem	-0.03	0.05	-0.60	0.55	-0.03	0.05	-0.60	0.55
Unal	243	Day length	-0.22	0.15	-1.45	0.15	0.12	0.09	1.38	0.17
	243	Dia_stem	-0.01	0.03	-0.23	0.82	-0.01	0.03	-0.19	0.85
Beaupre	250	Day length	-0.20	0.42	-0.48	0.63	0.12	0.25	0.48	0.63
	250	Dia_stem	0.07	0.02	3.07	0.00	0.07	0.02	3.07	0.00
Fritzy P.	250	Day length	-0.07	1.09	-0.06	0.95	0.03	0.64	0.05	0.96
	250	Dia_stem	0.01	0.05	0.23	0.82	0.01	0.05	0.23	0.82
Raspalje	250	Day length	0.22	0.34	0.63	0.53	-0.13	0.20	-0.62	0.54
	250	Dia_stem	0.03	0.03	1.16	0.25	0.03	0.03	1.16	0.25
Trichobel	250	Day length	-0.09	0.69	-0.13	0.90	0.05	0.41	0.13	0.89
	250	Dia_stem	0.00	0.05	0.08	0.93	0.00	0.05	0.08	0.93
Unal	250	Day length	-0.15	0.17	-0.89	0.37	0.08	0.10	0.84	0.40
	250	Dia_stem	0.03	0.03	0.93	0.35	0.03	0.03	0.95	0.34
Beaupre	257	Day length	-0.12	0.16	-0.72	0.47	0.07	0.10	0.73	0.46
	257	Dia_stem	0.03	0.01	2.66	0.01	0.03	0.01	2.66	0.01
Fritzy P.	257	Day length	-1.51	1.03	-1.47	0.14	0.89	0.60	1.47	0.14
	257	Dia_stem	0.00	0.05	-0.04	0.97	0.00	0.05	-0.05	0.96
Raspalje	257	Day length	0.37	0.24	1.53	0.12	-0.22	0.14	-1.53	0.13
	257	Dia_stem	0.03	0.03	1.14	0.25	0.03	0.03	1.15	0.25
Trichobel	257	Day length	-0.15	0.77	-0.20	0.84	0.09	0.45	0.20	0.84
	257	Dia_stem	-0.01	0.05	-0.12	0.90	-0.01	0.05	-0.12	0.90
Unal	257	Day length	0.10	0.12	0.83	0.40	-0.05	0.06	-0.87	0.39
	257	Dia_stem	0.05	0.02	2.18	0.03	0.05	0.02	2.19	0.03
Beaupre	264	Day length	-0.05	0.09	-0.56	0.58	0.03	0.05	0.57	0.57
	264	Dia_stem	0.01	0.01	1.85	0.06	0.01	0.01	1.85	0.06
Fritzy P.	264	Day length	-0.52	0.83	-0.62	0.53	0.30	0.49	0.63	0.53

		264	Dia_stem	-0.05	0.04	-1.25	0.21	-0.05	0.04	-1.25	0.21
	Raspalje	264	Day length	0.20	0.19	1.05	0.29	-0.12	0.11	-1.05	0.29
		264	Dia_stem	0.02	0.02	1.07	0.28	0.02	0.02	1.08	0.28
	Trichobel	264	Day length	-0.76	0.83	-0.92	0.36	0.45	0.48	0.93	0.35
		264	Dia_stem	0.03	0.05	0.55	0.58	0.03	0.05	0.55	0.58
	Unal	264	Day length	0.01	0.08	0.10	0.92	0.00	0.04	-0.10	0.92
		264	Dia_stem	0.01	0.02	0.45	0.65	0.01	0.02	0.45	0.65
Budburst 2015	Beaupre	83	Day length	NA	NA	NA	NA	NA	NA	NA	NA
		83	Dia_stem	NA	NA	NA	NA	NA	NA	NA	NA
	Fritzy P.	83	Day length	0.98	0.75	1.31	0.26	-0.57	0.44	-1.29	0.27
		83	Dia_stem	0.00	0.03	-0.10	0.92	0.00	0.03	-0.10	0.92
	Raspalje	83	Day length	-0.01	0.05	-0.11	0.92	0.00	0.03	0.12	0.92
		83	Dia_stem	0.00	0.01	0.12	0.90	0.00	0.01	0.12	0.90
	Trichobel	83	Day length	0.61	0.42	1.47	0.15	-0.36	0.25	-1.47	0.15
		83	Dia_stem	-0.01	0.03	-0.49	0.63	-0.01	0.03	-0.48	0.63
	Unal	83	Day length	-0.01	0.02	-0.81	0.42	0.01	0.01	0.83	0.41
		83	Dia_stem	0.00	0.00	-0.56	0.57	0.00	0.00	-0.57	0.57
	Beaupre	90	Day length	-0.03	0.02	-1.44	0.15	0.02	0.01	1.43	0.15
		90	Dia_stem	0.00	0.00	0.59	0.55	0.00	0.00	0.59	0.55
	Fritzy P.	90	Day length	0.33	0.56	0.59	0.56	-0.19	0.33	-0.58	0.57
		90	Dia_stem	0.06	0.03	2.29	0.03	0.06	0.03	2.29	0.03
	Raspalje	90	Day length	0.36	0.17	2.08	0.17	-0.21	0.10	-2.08	0.17
		90	Dia_stem	0.00	0.02	0.01	0.99	0.00	0.02	0.02	0.98
	Trichobel	90	Day length	0.08	0.66	0.13	0.91	-0.05	0.38	-0.12	0.92
		90	Dia_stem	0.06	0.03	1.71	0.09	0.06	0.03	1.71	0.09
	Unal	90	Day length	-0.08	0.07	-1.15	0.25	0.04	0.04	1.13	0.26
		90	Dia_stem	-0.01	0.02	-0.83	0.41	-0.01	0.02	-0.81	0.42
	Beaupre	97	Day length	-0.04	0.07	-0.60	0.61	0.03	0.04	0.61	0.61
		97	Dia_stem	0.00	0.01	0.78	0.44	0.00	0.01	0.78	0.44
	Fritzy P.	97	Day length	-0.40	0.84	-0.47	0.70	0.23	0.49	0.48	0.69
		97	Dia_stem	0.00	0.02	0.17	0.87	0.00	0.02	0.17	0.87
	Raspalje	97	Day length	0.28	0.22	1.26	0.21	-0.17	0.13	-1.27	0.21
		97	Dia_stem	0.02	0.02	0.89	0.38	0.02	0.02	0.89	0.37

Trichobel	97	Day length	-0.17	0.56	-0.30	0.79	0.10	0.33	0.31	0.78
	97	Dia_stem	-0.02	0.03	-0.55	0.58	-0.02	0.03	-0.55	0.58
Unal	97	Day length	-0.06	0.07	-0.87	0.42	0.04	0.04	0.91	0.40
	97	Dia_stem	-0.01	0.02	-0.66	0.51	-0.01	0.02	-0.68	0.50
Beaupre	104	Day length	-0.26	0.25	-1.06	0.29	0.15	0.15	1.06	0.29
	104	Dia_stem	-0.01	0.02	-0.64	0.53	-0.01	0.02	-0.64	0.53
Fritzy P.	104	Day length	-0.04	0.69	-0.05	0.96	0.02	0.41	0.05	0.96
	104	Dia_stem	0.09	0.03	2.54	0.01	0.09	0.03	2.54	0.01
Raspalje	104	Day length	0.32	0.31	1.03	0.30	-0.19	0.18	-1.04	0.30
	104	Dia_stem	-0.03	0.03	-0.86	0.39	-0.03	0.03	-0.86	0.39
Trichobel	104	Day length	-0.21	0.97	-0.22	0.85	0.13	0.57	0.22	0.85
	104	Dia_stem	-0.03	0.04	-0.73	0.47	-0.03	0.04	-0.73	0.47
Unal	104	Day length	-0.07	0.18	-0.38	0.72	0.04	0.10	0.40	0.71
	104	Dia_stem	-0.03	0.03	-1.06	0.29	-0.03	0.03	-1.07	0.29
Beaupre	111	Day length	-0.64	0.42	-1.52	0.13	0.38	0.25	1.53	0.13
	111	Dia_stem	-0.04	0.03	-1.27	0.21	-0.04	0.03	-1.27	0.21
Fritzy P.	111	Day length	NA	NA	NA	NA	NA	NA	NA	NA
	111	Dia_stem	NA	NA	NA	NA	NA	NA	NA	NA
Raspalje	111	Day length	0.17	0.37	0.45	0.66	-0.10	0.22	-0.45	0.65
	111	Dia_stem	-0.07	0.04	-1.71	0.09	-0.07	0.04	-1.71	0.09
Trichobel	111	Day length	0.00	0.12	-0.02	0.99	0.00	0.07	0.02	0.99
	111	Dia_stem	-0.01	0.01	-1.21	0.23	-0.01	0.01	-1.21	0.23
Unal	111	Day length	-0.06	0.28	-0.22	0.83	0.04	0.16	0.23	0.83
	111	Dia_stem	-0.05	0.04	-1.15	0.25	-0.05	0.04	-1.15	0.25
Beaupre	118	Day length	-0.46	0.38	-1.19	0.24	0.27	0.23	1.20	0.23
	118	Dia_stem	-0.02	0.03	-0.75	0.46	-0.02	0.03	-0.75	0.45
Fritzy P.	118	Day length	NA	NA	NA	NA	NA	NA	NA	NA
	118	Dia_stem	NA	NA	NA	NA	NA	NA	NA	NA
Raspalje	118	Day length	-0.01	0.23	-0.06	0.96	0.01	0.14	0.06	0.96
	118	Dia_stem	-0.02	0.02	-1.31	0.20	-0.02	0.02	-1.31	0.20
Trichobel	118	Day length	-0.10	0.36	-0.28	0.78	0.06	0.21	0.27	0.79
	118	Dia_stem	0.00	0.02	0.15	0.88	0.00	0.02	0.15	0.88
Unal	118	Day length	-0.05	0.12	-0.40	0.71	0.03	0.06	0.41	0.70

	118	Dia_stem	-0.03	0.02	-1.26	0.21	-0.03	0.02	-1.28	0.21
Beaupre	125	Day length	-0.25	0.29	-0.84	0.48	0.15	0.17	0.85	0.48
	125	Dia_stem	-0.02	0.02	-0.73	0.48	-0.02	0.02	-0.73	0.48
Fritzy P.	125	Day length	NA	NA	NA	NA	NA	NA	NA	NA
	125	Dia_stem	NA	NA	NA	NA	NA	NA	NA	NA
Raspalje	125	Day length	0.04	0.03	1.48	0.14	-0.02	0.02	-1.48	0.14
	125	Dia_stem	-0.01	0.00	-4.03	0.00	-0.01	0.00	-4.02	0.00
Trichobel	125	Day length	NA	NA	NA	NA	NA	NA	NA	NA
	125	Dia_stem	NA	NA	NA	NA	NA	NA	NA	NA
Unal	125	Day length	-0.02	0.04	-0.55	0.62	0.01	0.02	0.53	0.63
	125	Dia_stem	-0.01	0.01	-0.90	0.37	-0.01	0.01	-0.89	0.38

Reference

1. Kuznetsova A, Brockhoff PB, Christensen RHB. "lmerTest Package: Tests in Linear Mixed Effects Models.". Journal of Statistical Software. 2017;82(13):1-26. doi: doi: 10.18637/jss.v082.i13
